# Calcium channel gating

**DOI:** 10.1007/s00424-018-2163-7

**Published:** 2018-06-27

**Authors:** S. Hering, E.-M. Zangerl-Plessl, S. Beyl, A. Hohaus, S. Andranovits, E. N. Timin

**Affiliations:** Department of Pharmacology and Toxicology, University of Vienna, Althanstrasse 14, 1090 Vienna, Austria

**Keywords:** Calcium channel, Gating, Molecular modeling, Voltage sensor

## Abstract

Tuned calcium entry through voltage-gated calcium channels is a key requirement for many cellular functions. This is ensured by channel gates which open during membrane depolarizations and seal the pore at rest. The gating process is determined by distinct sub-processes: movement of voltage-sensing domains (charged S4 segments) as well as opening and closure of S6 gates. Neutralization of S4 charges revealed that pore opening of CaV1.2 is triggered by a “gate releasing” movement of all four S4 segments with activation of IS4 (and IIIS4) being a rate-limiting stage. Segment IS4 additionally plays a crucial role in channel inactivation. Remarkably, S4 segments carrying only a single charged residue efficiently participate in gating. However, the complete set of S4 charges is required for stabilization of the open state. Voltage clamp fluorometry, the cryo-EM structure of a mammalian calcium channel, biophysical and pharmacological studies, and mathematical simulations have all contributed to a novel interpretation of the role of voltage sensors in channel opening, closure, and inactivation. We illustrate the role of the different methodologies in gating studies and discuss the key molecular events leading CaV channels to open and to close.

**Electronic supplementary material:**

The online version of this article (10.1007/s00424-018-2163-7) contains supplementary material, which is available to authorized users.

## Introduction

The plateau of the cardiac action potential, contraction of muscle cells, generation of pace maker potentials, the release of hormones and neurotransmitters, sensory functions, and gene expression are all mediated by fine-tuned calcium entry through voltage-dependent calcium channels [27, 49, 96, 120]. Mutations in calcium channels that disturb the channel gating lead consequently to diseased states called calcium channelopathies (Table 1) [20, 94].

**Table 1:** Selected channelopathy mutations in S4 or pore-forming segments with known gating disturbances

Mutation/segment CaV	Pathology	Gating disturbance	Reference
R528H/IIS4 R1239H/IVS4 CaV1.1	Hypokalemic periodic paralysis	R528H: leftward shift of steady state inactivation R1239H: reduction of channel expression	Sipos et al. [86] Lehmann-Horn et al. [59]
R174W/IS4 CaV1.1	Malignant hyperthermia susceptibility	Reduction of calcium current Leftward shift of steady state activation	Carpenter et al. [26]
G406R/IS6 CaV1.2	Timothy syndrome	Deceleration (removal) of channel inactivation	Splawski et al. [89]
G403R/IS6^a^ I770M/IIS6^b^ CaV1.3	Adrenal aldosterone-producing adenomas	G403R: leftward shift of steady state activation, deceleration (removal) of inactivation I770M: leftward shift of steady state activation and inactivation	Scholl et al. [84]
G407R/IS6 A749G/IIS6^c^ CaV1.3	Autism spectrum disorders	G407R: deceleration of inactivation A749G: leftward shift of steady state activation and inactivation	Pinggera et al. [77]
V401L/ CaV1.3	Autism spectrum disorders and epilepsy	Leftward shift of steady state activation and inactivation, enhanced current density, reduction of inactivation	Pinggera et al. [78]
Insertion of a Glycine residue between residues 403^d^ and 404/IS6 CaV1.3	Bradycardia and congenital deafness	Non-conducting channels	Baig et al. [9]
G369D/IS6^e^ CaV1.4	Congenital Stationary Night Blindness Type 2	Leftward shift of steady state activation, deceleration of inactivation, removal of Ca^2+^ dependent inactivation	Hoda et al. [48]
I745T/IIS6 CaV1.4	Incomplete congenital stationary night blindness (CSNB2)	Leftward shift of steady state activation, deceleration of inactivation	Hemara-Wahanui et al. [44]
R192Q/1S4 Cav2.1	Familial hemiplegic migraine (FHM1)	Leftward shift of steady state activation	Mellitti et al. [67] Hans et al. [43] Kraus et al. [57]
V714A/IIS6 I1811L/IVS6 Cav2.1	Familial hemiplegic migraine (FHM1)	V714A: leftward shift of steady state activation I1811L: deceleration of inactivation	Hans et al. [43] Kraus et al. [57]
R1715/IVS4 CaV3.1	Spinocerebellar ataxia	Rightward shift of steady state activation and inactivation	Morino et al. [69]

^a^G403 in CaV1.3 corresponds to G/A/G/A residue G432 in CaV1.2

^b^I770 in CaV1.3 corresponds to I745 in CaV1.4 causing incomplete congenital stationary night blindness [44]

^c^A749 corresponds to the G/A/G/A residue A780 in CaV1.2

^d^G403 corresponds to G/A/G/A residue G432 in CaV1.2

^e^G369D corresponds to G432 in Cav1.2

Calcium channels can be subdivided into high-voltage activated (CaV 1.1–1.4, CaV2.1–2.3 and low-voltage activated CaV3.1–3.3 channels [5, 25, 70, 74–76, 119]. Their different voltages of activation, kinetics, and pharmacological properties are predominantly determined by their pore-forming α1 subunits but additionally affected by associated auxiliary subunits [21, 22, 45, 81, 120]. Wu et al. were the first to report full-length structures of CaV α1 subunits (rabbit CaV1.1 complex) composed of an α1S, an extracellular α2δ, an intracellular β, and a transmembrane γ subunit with an overall resolution of 4.2 and 3.6 Å [106, 107].

In this review, we focus on the molecular determinants of voltage-dependent opening and closure of the highly homologous CaV1.2 (for mechanism of calcium-dependent inactivation see i.e. Ben-Johny et al. [11]). Quantification of current kinetics and steady state activation of CaV1.2 by Beyl et al. [16] revealed the following sequence of events: At rest, the voltage sensors (VSs) are pulled into a down position by the electrical field. In their down state, the VSs lock the channel in its closed state. Membrane depolarization releases the VSs, resulting in their almost voltage-independent upward movement, which in turn releases the closed channel gates. The pore at first stays closed until the S6 gates disengage and the channel opens. Channel opening and inactivation are enabled when all four S4 segments have left their resting position (see also Horn et al. [51]). Here, we discuss how each of the four S4 segments is linked to channel activation and inactivation and propose a refined gating model for CaV1.2.

## Architecture of the CaV1.1 and CaV1.2 channel

**Fig. 1:**
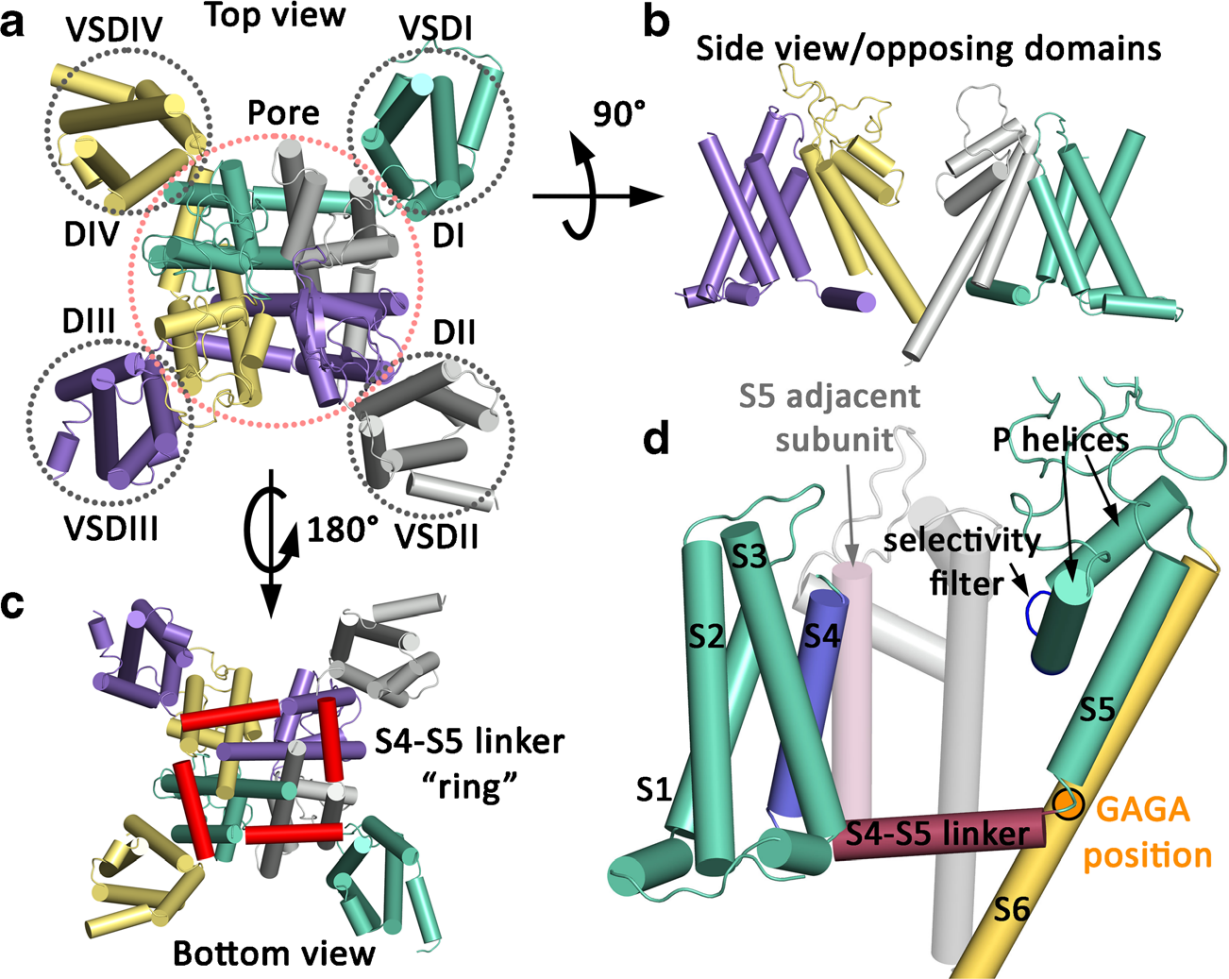
Model of a CaV1.2 α1 subunit (based on the cryo-EM structure of CaV1.1 by Wu et al. [106, 107]). CaV1.1 and CaV1.2 are highly homologous (see sequence alignment in Supplementary Fig. 1). The helices are represented as cylinders. Domains I to IV are shown in green, gray, purple, and yellow, respectively. **a** The top view arrangement of the channel with the voltage-sensing domains (VSDI to VSDIV) and pore domain. **b** The side view shows the structural elements of opposing domains. The bundle-crossing region at the lower third of the S6 segments forms the activation gate. **c** The bottom view with a highlight on the S4–S5 linker ring (in red). The S4–S5 linker helices run in parallel to the intracellular side of the membrane. **d** One domain of the α subunit of the voltage-gated calcium channel. S1 to S3 (green) and S4 (blue) represent the voltage-sensing domain. Segment S5, the P-helices (green), the selectivity filter (dark blue), and the S6 (yellow) form the pore domain. S4 is connected to the S5 via the S4–S5 linker (red). The S4 is further in close proximity to the S5 of the adjacent subunit (light pink). The S6 as well as the P-helices of the adjacent subunit are shown in light gray. The G/A/G/A position is highlighted in orange (Depil et al. [36]), close to the loop between the S4–S5 linker and the S5 helix

**Fig. 2:**
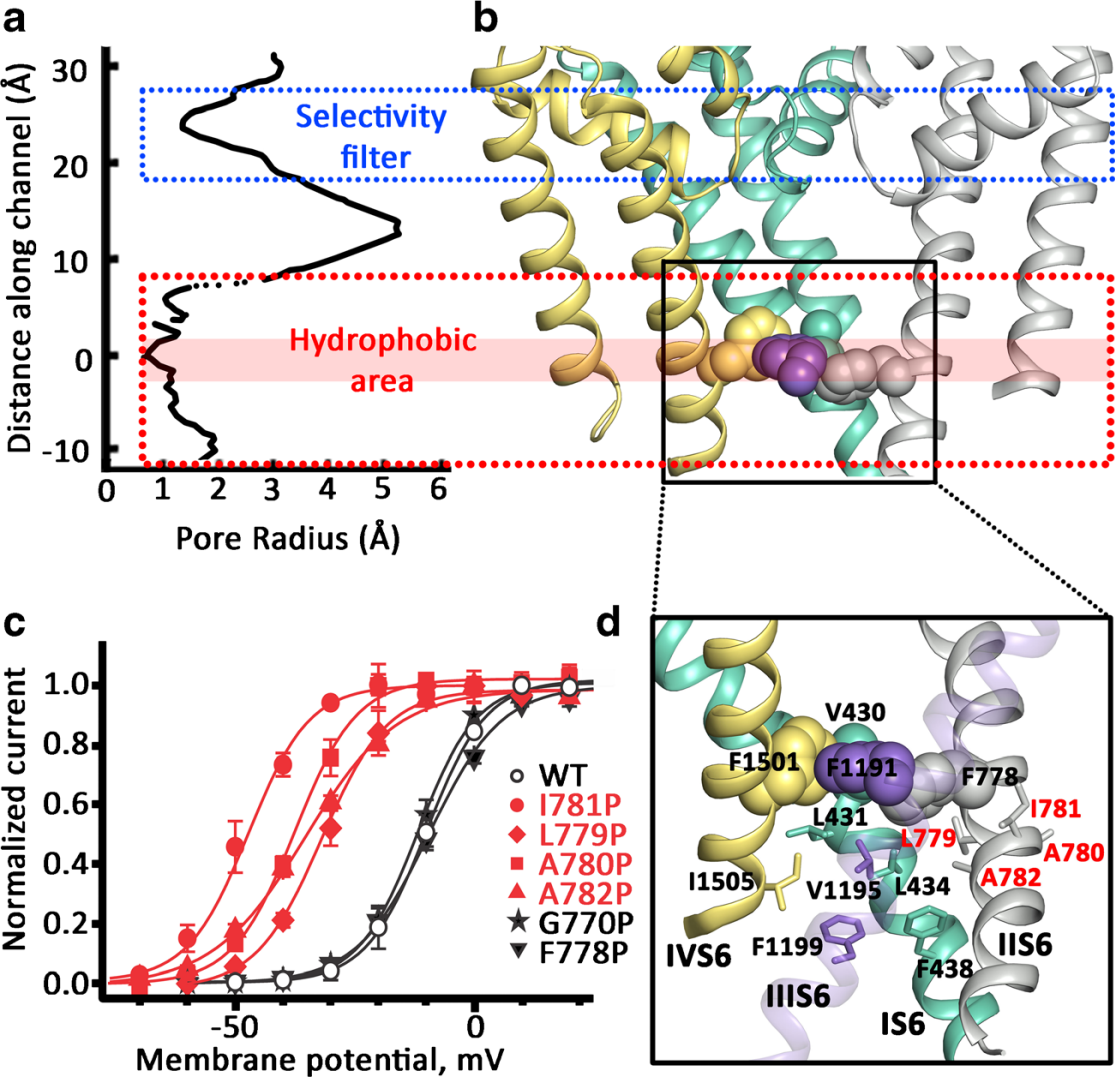
Location of the activation gate: hydrophobic residues form a sealing region at the intracellular gate. **a** The permeation path of the pore domain (modified from Wu et al. [106]). The pore regions including S6 and selectivity filter between CaV1.1 and CaV1.2 are highly homologous (see Fig. S1 in supplemental materials). The figure displays the pore radii of CaV1.1 along the pore. The closest part of the hydrophobic area (**a**, position around 0) formed by V430, F778, F1191, and F1501 is emphasized with a red bar. The helix bundle crossing region is highlighted with a red dotted box. The blue dotted box marks the selectivity filter. **b** Cartoon representation of the pore domain. The third domain is omitted for clarity. Domain 2 (DII) is colored in gray, DIII in purple, and DIV in yellow. The dotted rectangles from **a** are extended to this figure to show the corresponding areas in the protein. The occluding residues V430, F778, F1191, and F1501 from the cavity facing the intracellular side are represented as spheres. **c** Effects of proline substitutions in positions F778–A782 and G770 on the voltage dependence of CaV1.2 activation. Solid lines represent fits to Boltzmann functions. Mutations causing large shifts and corresponding activation curves are shown in red. This research was originally published in the *Journal of Biological Chemistry*. Hohaus et al. [50]. **d** Detailed view on the helix bundle crossing region. The S6 helices are represented as cartoon with the third domain set transparent for clarity. Domain DI, DII, DIII, and DIV are colored in green, gray, purple, and yellow, respectively. The occluding residues V430, F778, F1191, and F1501 from the cavity side are represented as spheres. Please pay attention to the extended hydrophobic cluster of residues below the narrowest occluding positions that are represented as sticks. It is tempting to speculate that the hydrophobic cluster contributes to closed state stability to the activated not open state A (Fig. 3, see also del Camino et al. [24])

The CaV1.1 α subunit is composed of four domains concatenated in a single polypeptide chain. Each domain consists of a voltage-sensing domain (helices S1 to S4) and the pore-forming module (helices S5 and S6, the P1 and P2 helices, Fig. 1). The selectivity filter, located at the extracellular side of the transmembrane domain (TMD), contains the EEEE locus [39, 106, 107, 111]. The selectivity filter extends to the channel cavity, which is surrounded by a tetrameric arrangement of the S5 and S6 helices (see Fig. 1). The S6 helices form the activation gate at the intracellular end of the TMD [108]. In the closed state, the pore-lining S6 helices converge at the intracellular side to obstruct ion permeation. Modeling of CaV1.2 based on the CaV1.1 crystal structure by Wu et al. [106] shows that the cavity is occluded towards the intracellular side starting at the amino acids V430, F778, F1191, and F1501 from IS6 to IVS6, (see Fig. 2), including a hydrophobic region extending towards the intracellular side.

Upon opening, the intracellular ends of the S6 helices diverge from one another and thereby open wide enough to enable ions to pass. Computational and experimental studies on potassium and sodium channels propose a pivoting motion of the S6 helix, starting at a hinge-point in the middle of the helix [38, 62, 64, 83, 116, 122]. Although it is widely agreed that such a movement takes place, the extent of channel opening at the activation gate varies among different published structures [4, 7, 8, 10, 42, 60, 66, 97, 115, 123]. A glycine that is frequently found at this hinge-point position (which allows a wider range of phi angles) provides bending flexibility [23, 37, 52, 64, 101]. However, the S6 mutation G770P in domain II of CaV1.2 (corresponding to the location of “gating-hinges” in MthK (G83, [54]) and NaChBak (G219, [122])) affects neither the current kinetics nor the position of the activation curve, which suggests that the mechanism is distinct from potassium channel gating (see Fig. 2c, [50]).

Individual VSDs are composed of four membrane spanning α-helices. The actual VSs are the S4 segments, in the case of the CaV1.1 structure a 3_10_ helix [106]. These helices contain positively charged arginine or lysine residues at every third or fourth position [73, 85, 104–107, 110, 121]. Consistent with the crystallography and cryo-EM environment, the S4 segment of the VSs of all reported calcium, sodium, and potassium voltage-gated ion channel structures is in the upstate. However, the “levels” of the upstates do vary in different structures, within some structures also between different domains. These “levels” are measured by the number of positively charged amino acids that are above a bulky hydrophobic residue of the so-called charge transfer center (CTC). The CTC is formed by conserved negative or polar residues as well as the highly conserved occluding bulky hydrophobic residue on the S2 and an invariant aspartate residue on the S3 [18, 79]. These residues, in addition to another negative or polar residue on S2, are important for sequential charge-charge interactions to the positively charged amino acids in S4 to catalyze its transmembrane movement [28, 33, 34, 61, 100, 102, 114]. In the latest CaV1.1 structure [106], the electron density map quality of the VSDIII does not allow assignment of the residues on S3 and S4. The authors propose the VSs are in a depolarized or upstate. In VSDI, four charges, R1 to R4, are above the occluding hydrophobic residue, in this case a phenylalanine, of the CTC. In VSDII, only three charges, R2 to R4, are above the occluding phenylalanine, suggesting induvidual and asynchronous movements of S4 segments.

The S4 and S5 helices are connected via the S4–S5 linker (see Fig. 1c, d)—a helix which runs parallel to the intracellular side of the membrane and almost surrounds the pore domain at its intracellular side (see Fig. 1c). Each voltage-sensing domain is adjacent to the neighboring pore domain (e.g. see [35]), with the S4 of one domain forming hydrophobic interactions with the S5 of the adjacent domain (Fig. 1d). This clockwise assembly not only allows the VSDs to influence the gate of their own domains but potentially also the neighboring domains [110]. The impact of this interaction is currently unknown. In comparison, in Kv11.1 [104] and Kv10.1 [105], the voltage-sensing S4 segments are connected to the pore of the same domain by a short linker loop and every S4 has hydrophobic interactions with only the S5 of the same domain.

## Localization of the activation gate

In the closed state, the pore-lining S6 helices converge at the intracellular side and obstruct ion permeation. A retinal disorder caused by a point mutation in segment IIS6 (I745T) of the CaV1.4 α1 subunit yielded insights into the gating mechanism [44]. Figure 2 illustrates that mutation I781T in IIS6 of the CaV1.2 α1 subunit (corresponding to I745T in CaV1.4) is located within the S6 helix bundle-crossing region. This threonine substitution (or mutation to proline) shifted the activation curves to the left [50], indicating reduced stability of the closed state and/or increased stability of the open state. Residue I781 is part of a cluster of hydrophobic residues, where proline substitutions cause prominent left shifts of the activation curve and marked slowing in current in the lower third of segment IIS6 (L779-A782, called LAIA motif, Fig. 2, [50]). A similar gating sensitive hydrophobic stretch of amino acids (VAVIM motif) in the lower third of segment IVS6 was identified in CaV2.3 by Raybaud et al. [82] (see also Zhao et al. [122] for slow gating phenotypes in NaChBak S6 mutants). These functional data are in agreement with the structural information. As illustrated in Fig. 2d, residues in the lower third of the S6 gates form a tight hydrophobic cluster. This cluster seals off the inner cavity from the intracellular environment. Disturbance in this region (e.g., by proline-induced bending of the helix) might induce small structural changes leading to wetting of this area, which would destabilize the closed state (Fig. 2d).

Interestingly, mutating residue F778 that was highlighted as one of the “pore-occluding S6 residues” (V430, F778, F1181, and F1501 [50]) did not affect the activation curve of CaV1.2 and thus had no effect on closed-state stability (Fig. 2d).

Taken together, functional and structural studies support a key role of I781 and three neighboring residues (L779, A780, and A782) in gating of the CaV1.2 channel, indicating that this region forms part of the channel’s activation gate (for corresponding gating determinants in CaV2.3, see Raybaud et al. [82]). Interactions with neighboring residues in all four S6 segments apparently contribute to stabilization of the “activated not open” channel state (see del Camino et al. 2005 [24]; see also Fig. 5b for illustration of amino acid properties in gating). A role of A780 in coupling the VS and channel pore will be discussed below (Figs. 5 and 10).

## The four-state gating cycle of CaV activation

**Fig. 3:**
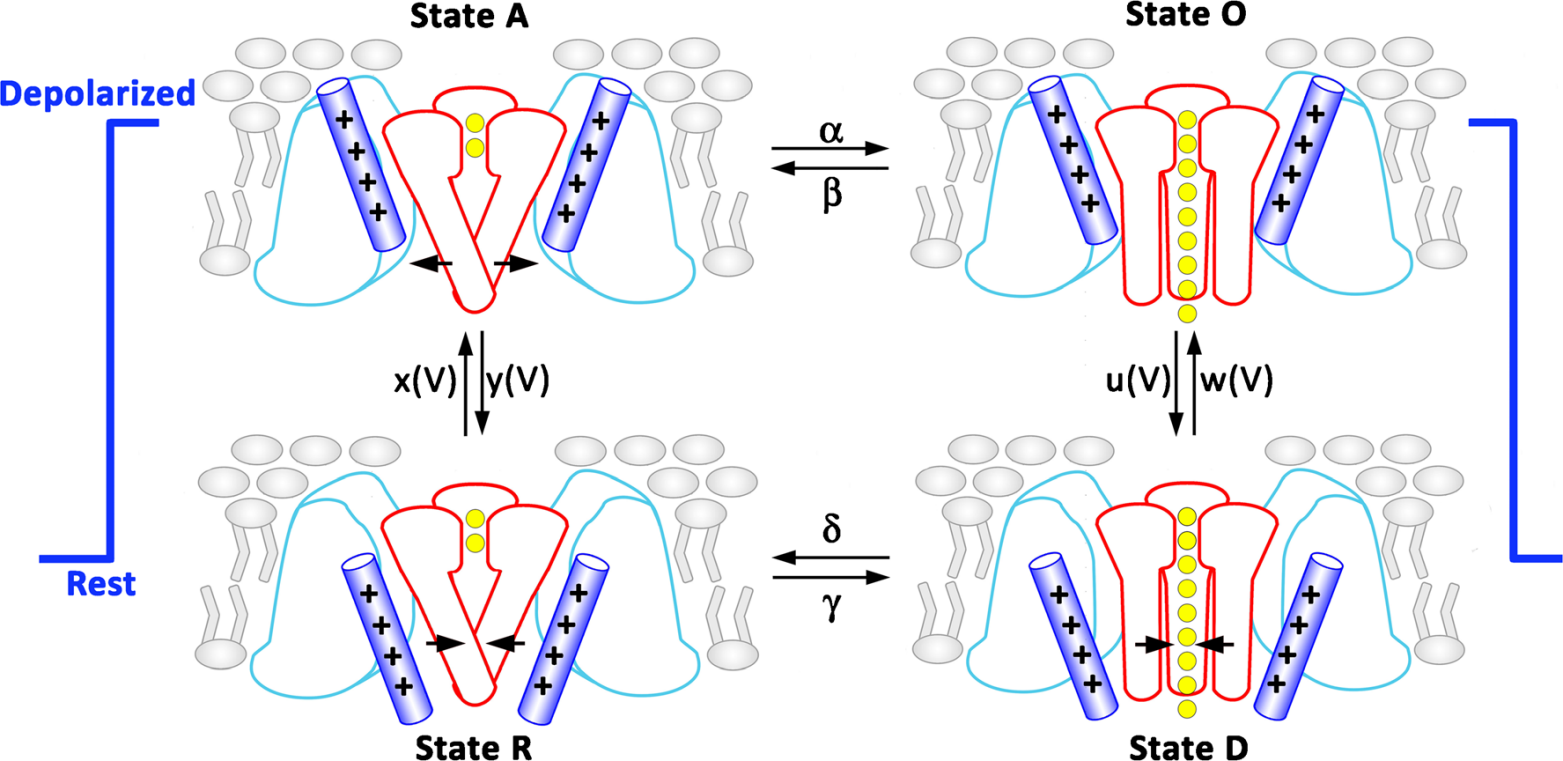
State transitions during activation (modified after Beyl et al. [16]) Activation gating is determined by two functionally separate processes: a voltage-sensing mechanism (++++) and the conducting pore. Each functional unit can dwell in two states: the VS in the resting (down) and activated (up) states and the pore in the open or closed states. The entire molecule therefore dwells in 2 × 2 = 4 states: R, pore is closed and voltage-sensing mechanism locks the pore; A, voltage-sensing mechanism is activated and releases the pore, which, however, remains closed; O, the pore is open; D, the deactivated voltage-sensing mechanism is in the down position while the pore is still open. Rate constants of the pore opening and closure (*α*, *β*, *γ*, *δ*) are assumed to be voltage-independent. Rate constants of voltage-sensing mechanism (*x*, *y*, *u*, and *w*) are voltage dependent

As illustrated in Fig. 3, VSs can dwell in two states, resting (down) and activated (up) states, and in addition, the pore has two states: open or closed [16]. Correspondingly, the channel dwells in 2 × 2 = 4 states (Fig. 3 adapted from Beyl et al. [16]):

- R (resting closed state): pore is closed and VSs lock the pore
- A (activated closed state): pore is closed, but the VSs are in their “up” position
- O (activated open state): pore is open and the VSs are in the “up” position
- D (deactivated open state): pore is still open though the VSs are in their down position

The VSs move in response to a depolarization from “resting down” to an “activated up” position [2, 3, 90, 91, 112, 113]. The channel thus switches from state R to A with voltage-dependent rate constants *x*(*V*) and *y*(*V*). When the VSs are in the activated position, the pore opens and closes with the voltage-independent rate constants *α* and *β* (channel transfers between states A and O). When the channel is in the open state, a hyperpolarization first induces a downward movement of the VS (transition O ↔ D) which is followed by pore closure (transition D ↔ R).

This model differs from traditional kinetic models of activation [117, 118] in two main aspects: (i) a new state D was introduced, where the channel is deactivated but still open, and (ii) multiple intermediate transitions are lumped together in a one-step transition R ↔ A.

Although the model outlined in Fig. 3 fits most of the functional data on calcium current activation, it is still a simplification. Indeed, activation of the VSs comprising four charge-carrying S4 segments can be imagined as a multi-exponential process. During activation, each of the four VSs can dwell in either the resting (down) or activated (up) state, resulting in 2^4^ = 16 individual combinations (IS4–up/IIS4–down/IIIS4–down/IVS4–down, IS4–up/IIS4–up/IIIS4–down/IVS4–down, etc.). The activation of CaV1.2 is, however, predominantly mono-exponential and lumping together potential transitions between intermediate states seems justified. This model differs from descriptions of activation of the potassium channels, where the current develops with a significant delay (Cole–Moore delay) resulting from sequential transitions of individual VSs [72, 87].

The advantage of this four-state model is that it enables the quantification of the VS activation and pore opening from current kinetics with widely accepted constraints: (i) rate constants of the pore opening α and β are voltage-independent and (ii) the voltage dependence of the rate constants of activation is described by Eyring functions: $x(V)={x}_0\cdot \exp \left(\frac{V}{k_x}\right)$ and $y(V)={y}_0\cdot \exp \left(-\frac{V}{k_y}\right)$. Application of the inverse problem approach yielded the parameters of the model that, in turn, allowed an interpretation of changes in steady state and kinetics of activation induced by mutations either on the pore or in VS segments [16].

## State R

CaV channels remain in the R state at hyperpolarizing voltages (Fig. 3). In this conformation, the channel gates are closed. S4 segments in their down position lock the gate and prevent opening. Structural and functional studies revealed that closed channel gates prevent not only the inflow of calcium ions but can also inhibit dissociation of drug molecules from binding sites within the channel pore [17].

**Fig. 4:**
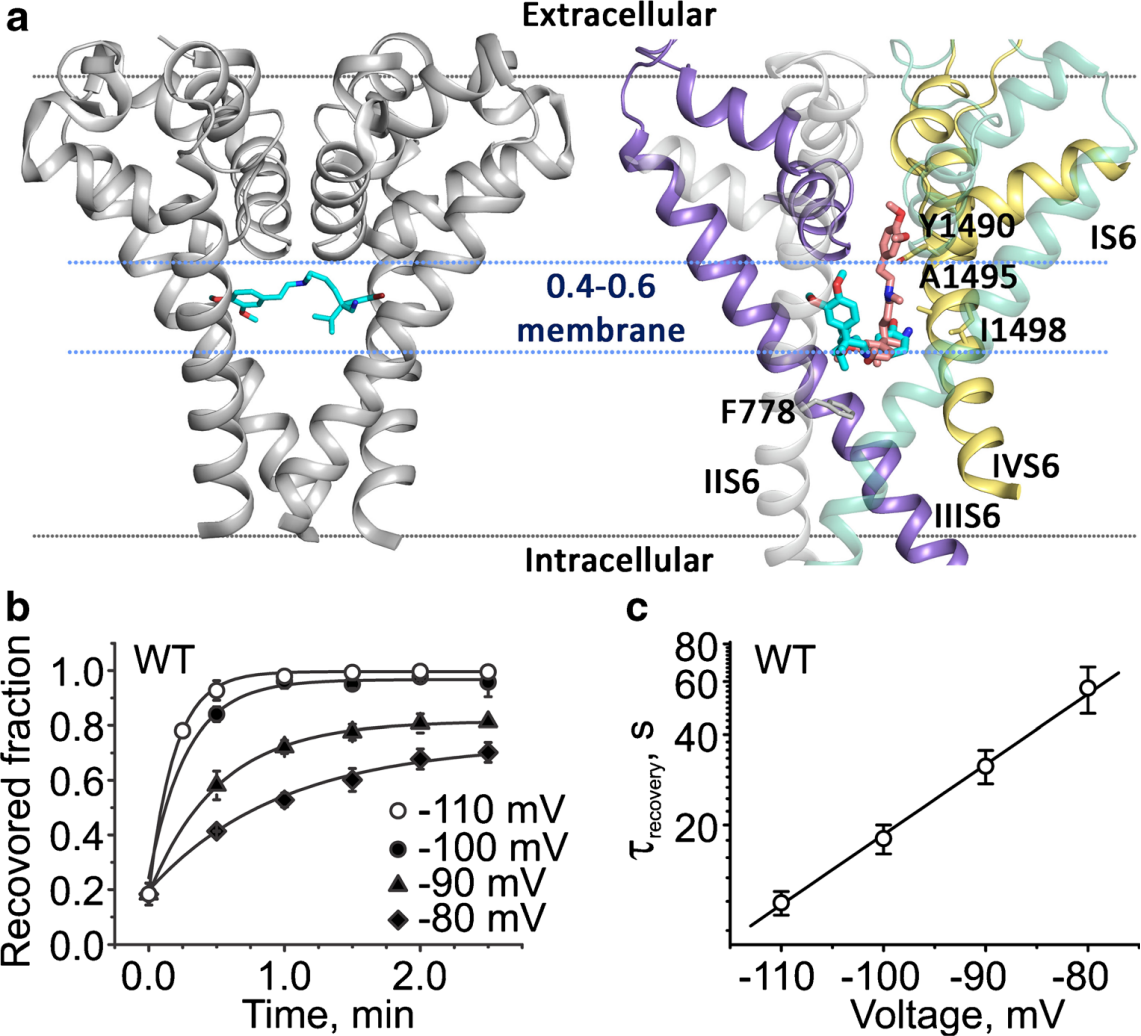
Closed channel gates trap a phenylalkylamine. **a** *Left*: Crystal structure of CaVAb in complex with Br-verapamil (pdb-code: 5kmh [98]). The P-helices and S6 helices are represented as cartoon. The Br-verapamil is shown as cyan sticks. The area spanned by the membrane is shown in gray. The region between the two blue dotted lines corresponds to 0.4–0.6 fraction of the membrane. *Right*: (−)qD888 docked into the cavity of the homology model of the CaV1.2 closed conformation. Domains 1 to 4 are colored in green, gray, purple, and yellow, respectively. Domains 1 and 2 are set transparent for better visibility. Compound (−)qD888 is shown as cyan and salmon sticks. The figure illustrates two high score poses. The previously identified putative binding determinants that are within 5 Å of the compound are represented as sticks [47]. The permanently charged nitrogen is located for both docking poses within the labeled fraction of 0.4–0.6 of the membrane potential. **b** Recovery of CaV1.2 from block by (−)qD888 (100 μM). (−)qD888 was applied to the intracellular side of the membrane and recovery from block was measured. Block was induced by a train of test pulses from − 80 to 10 mV and the holding potential subsequently switched to − 80, − 90, − 100, or − 110 mV. The fraction of recovered channels was measured after different time intervals. **c** Plot of the time constants of recovery versus voltage in semi-logarithmic coordinates revealed an exponential dependence with faster recovery at more negative voltages. Eyring analysis predicts a location of the charged locus of the PAA molecule close to the central pore region (the estimated fraction of the membrane potential affecting drug dissociation was 0.56, Beyl et al. [17]). For **c** and **d**: This research was originally published in the *Journal of Biological Chemistry*. Beyl et al. [17]. © the American Society for Biochemistry and Molecular Biology

Crystallographic analyses of phenylalkylamine binding to the bacterial homotetrameric CaVAb channel showed how a Br-verapamil is trapped in the central cavity by closed S6 gates [98] (Fig. 4a). Studies of use-dependent CaV1.2 inhibition by the permanently charged phenylalkylamine (−)devapamil ((−)qD888) revealed unrestricted access of this large molecule to its binding determinants in the open state [12, 17, 47]. Recovery from block was voltage dependent with faster channel unblock at hyperpolarized voltages. This supports a scenario where negative voltages “pull” positively charged (−)qD888 from its binding site on segments IIIS6 and IVS6 [94] through completely or partially closed gates [17]. From recovery kinetics, it was estimated that a membrane potential fraction of 0.56 affects drug dissociation (Fig. 4b, c; [17]). Assuming a quasi-linear distribution of the potential within the closed channel yields a localization of (−)qD888 within the central pore region. Together, these structural (Fig. 4a) and functional studies (Fig. 4b, c) confirm a binding site for phenylalkylamines within the central cavity.

## R ↔ A transition

During this transition, the S4 shuffles its charges above the CTC, thereby releasing the gate. Quantification of CaV1.2 kinetics in terms of the four-state model (Fig. 3) revealed strong voltage dependence of the downward transitions of S4 (rate constant y(V)) compared to weak voltage dependence of the upward movement (*x*(*V*)) [16]. This suggests that the membrane voltage is more efficient in pushing the CaV1.2 into the closed state R than “pulling” the gate open (see also Fedida and Hesketh [40]).

A scenario in which the S4 segments release the gates rather than pulling them open is also evident from other voltage-gated channels. For example, KCNH potassium channel constructs in which “S4 pulling” was excluded by splitting the S4–S5 linker, still opened in a voltage-dependent manner [63].

## State A

Studies on Shaker ILT mutants revealed that cysteines engineered into S6 gates are accessible mainly in an activated not open state which provides evidence for the existence of state A [24]. The available CaV1.1 structures with all four VSs presumably in an up position resemble this conformation (A) [98, 106, 107] (Figs. 1 and 3). The question arises: What keeps an unlocked gate structure shut?

A variety of S6 mutations in the bundle crossing region shift the activation curve (ΔV_0.5act_, Fig. 2c) in the hyperpolarizing direction (A) and decelerate current activation and deactivation (exemplified for I781P) indicating closed state destabilization and/or stabilization of the open channel conformation.

**Fig. 5:**
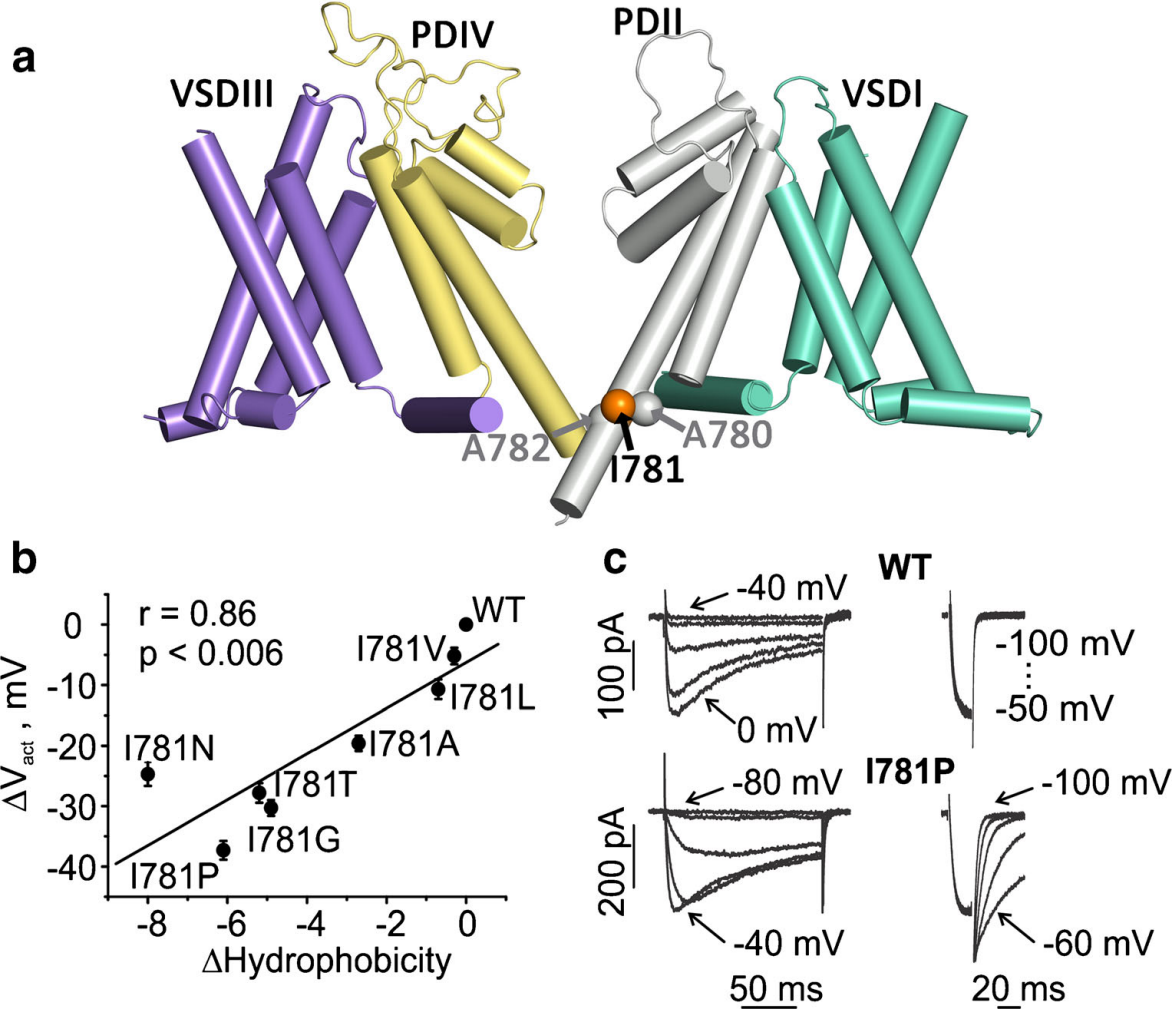
Hydrophobic interactions stabilize “activated not open” conformation: Midpoint shifts of the activation curve (ΔVact) correlate with changes in hydrophobicity in position I781 on IIS6. **a** The side view of the CaV1.2 homology model (see Fig. 1) shows the structural elements of opposing elements of the domains with the helices represented as cylinders. The VSDs of domain I and III are colored in green and purple, respectively. The pore-forming domains of domain 2 and 4 are colored in gray and yellow, respectively. Positions of I781 (as well as A780 and A782 from the L/A/I/A motive) are highlighted as orange and gray balls. **b** Amino acid substitutions in position I781 (corresponding to the channelopathy mutation I745T in CaV1.4, [44]) destabilize the closed conformation and stabilize the open conformation of CaV1.2. In other words, changes in hydrophobicity in position I781 predict the shifts of the activation curve (see also Beyl et al. [14] for the role of other amino acid descriptors). Figure from *Pflügers Archiv – European Journal of Physiology*. Beyl et al. [14]. © The Authors. **c** A leftward shift of the activation curve is accompanied by deceleration of kinetics. Left panel shows representative families of I_Ba_ through wild type (upper traces) and I781P mutant channel (lower traces). Membrane potentials (left) indicate the threshold of channel opening (− 40 mV in WT and − 80 mV in I781t) and voltages applied for tail current measurements (right). This research was originally published in the *Journal of Biological Chemistry*. Hohaus et al. [49]. © The American Society for Biochemistry and Molecular Biology

Insights into the molecular determinants of closed and open state stability can be obtained if we examine the relation between the physicochemical parameters of gate-forming amino acids (descriptors of residues in the bundle crossing region) and the shifts of the midpoints of the activation curve (ΔV_0.5act_, Fig. 5).

Hydrophobicity emerged as the leading determinant of closed gate stability in position 781 (illustrated in Fig. 5b). However, hydrophobic interactions [58] are not the only interactions that contribute to closed state stability. Combined descriptor analysis addressing several amino acid properties simultaneously can substantially improve the correlation indicating that a combination of different amino acid properties contributes to the stability of open or closed conformations (see [14]).

## A ↔ O transition

During continuous depolarization, the S6 gates disengage and CaV channels move from the activated (A) to the open state (O, Fig. 3). This disengagement of the bundle crossing region (concerted pore opening) is a dynamic process. Molecular dynamic simulations illustrate that S6 gates may flicker between engaged and free positions [53]. The concerted pore opening occurs when all four S6 gates disengage and are stabilized in an open position.

## State O

CaV1.2 remains in the open state during depolarization. There is evidence that the channel gates open wide enough to enable unrestricted access of a large molecule like (−)qD888 to its binding site in the cavity [17].

## State D, O↔ D, and D ↔ R transitions

Little is known about state D and corresponding transitions. State D was first postulated to describe the process of CaV channel deactivation (Fig. 3 in [16]). A three-state model (Rest ↔ Activated ↔ Open) failed to describe CaV1.2 current kinetics and in particular was unable to reproduce the acceleration of channel deactivation kinetics that is experimentally observed (see faster channel deactivation at hyperpolarized voltages illustrated by the bell shaped curves in Figs. 7c, 9f, and 10e). This discrepancy is caused by the slow O ↔ A transition, which is a rate-limiting step in a 3 state model and prevents faster deactivation at stronger hyperpolarization (see simulation in [16]).

Further evidence for state D came from simulation studies. Jensen et al. [53] used all-atom molecular dynamics simulations to observe the conformational changes in a potassium channel during closure. The deduced mechanistic model comprises a transition from an activated to a deactivated channel conformation where the VSs moved towards a down position while the pore is still open.

So far, there is no structural evidence for a calcium channel in state D. This is not surprising since in the crystallography and cryo-EM environment, no electrical field is applied. The deactivated state is expected to occur exclusively at hyperpolarized voltages after the channels have passed the open conformation. Thus, observations of state D remain a particular challenge and will require studies under hyperpolarized (i.e., physiologically relevant) conditions.

## Voltage sensors move at different rates

**Fig. 6:**
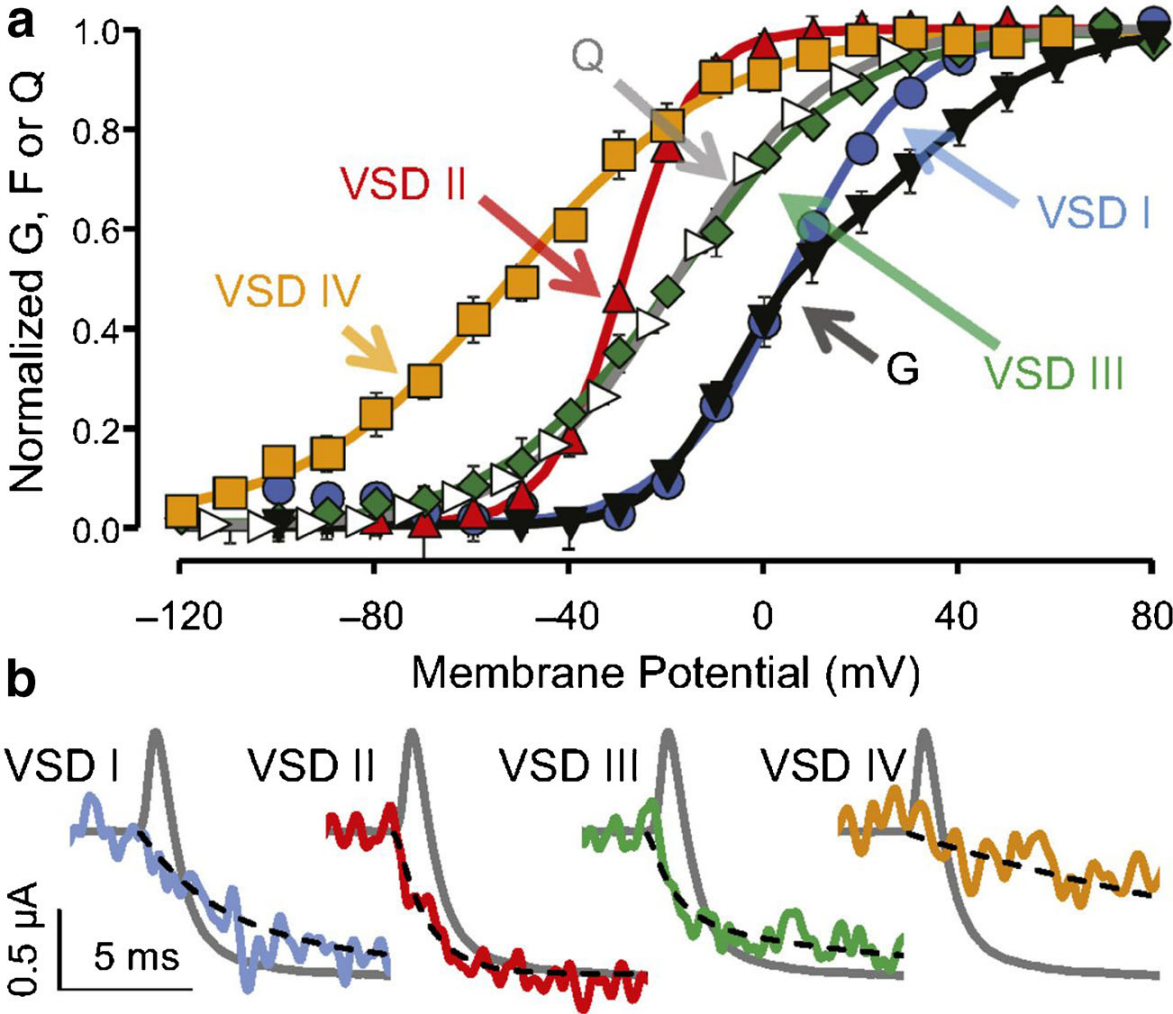
Fluorophore labeled voltage sensors exhibit distinct voltage dependence and kinetics. **a** Mean normalized conductance (G; black down-pointing triangle) and charge movement (Q; white right-pointing triangle) from WT channels and fluorescence (F) from VSDs I (blue circle), II (red up-pointing triangle), III (green diamond), and IV (yellow square). The curves are fits to single or (for G) the sum of two Boltzmann distributions. Error bars indicate ± SEM. **b** Representative membrane current (gray) from WT channels for a − 90– → 20–mV pulse, with superimposed F reported from VSD I (*τ*1 = 2.6 ms, 59%; *τ*2 = 8.1 ms), II (*τ*1 = 1.1 ms, 98%; *τ*2 = 20 ms), III (*τ*1 = 0.88 ms, 68%; *τ*2 = 9.2 ms), and IV (*τ* = 17 ms). The black dashed lines are exponential functions with the reported time constants. The sequence of activation for the CaV1.2 VSDs (half-time to maximum, *t*0.5) is VSD II (1.0 ms), III (1.4 ms), I (2.9 ms), and IV (11 ms). Figure and adapted subscript from *Proceedings of the National Academy of Sciences*. Pantazis et al. [70]. A gating scheme deduced from these experiments is illustrated in Fig. 12c

In order to track the conformational transitions of individual VSs, Pantazis et al. [71] labeled the extracellular flank of each S4 segment (IS4–IVS4) of the α1 subunit of CaV1.2 with a fluorophore serving as an optical reporter (Fig. 6) [71]. The resulting Δ*F*–*V* curves were interpreted to reflect conformational changes associated with individual S4 movements. As illustrated in Fig. 6, each S4 segment activates at different voltages. Pantazis et al. [71] demonstrated that voltage dependence and fast kinetic components in the activation of IIS4 and IIIS4 were compatible with the kinetics of the ionic currents (Fig. 6b). Making use of an allosteric gating model, the authors concluded that IIS4 and IIIS4 supply most of the energy for stabilization of the open state (Fig. 12c).

## Up-movement of IS4 (and IIIS4) is a rate-limiting state for pore opening

**Fig. 7:**
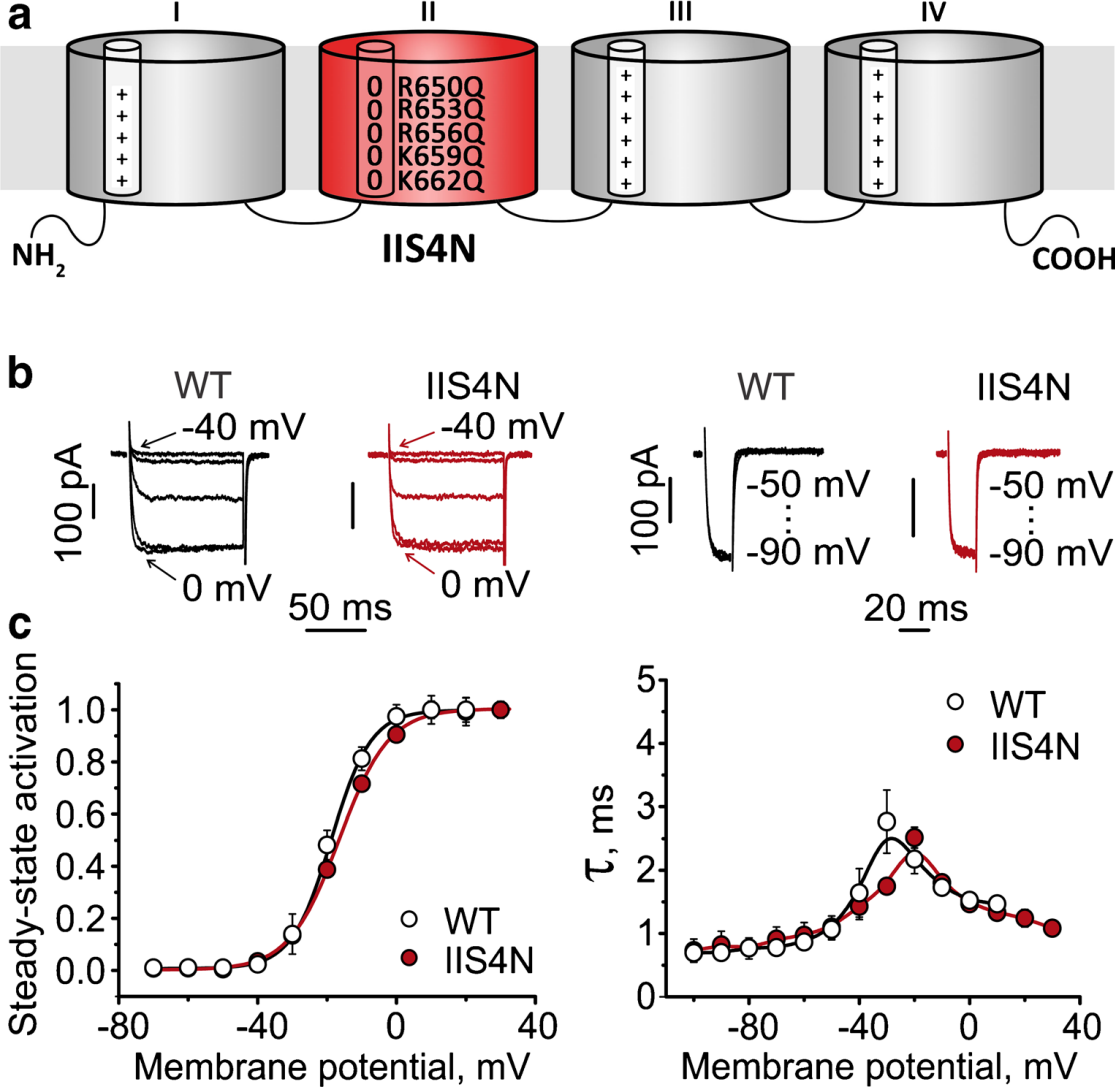
Role of IIS4 in activation of wild type CaV1.2: Neutralization of all charges (construct IIS4N) has no effects on activation gating. **a** Schematic representation of the α1 subunit. Domains are numbered from I to IV. The S4 helices of each domain are represented as small cylinders. Zeros stand for charge neutralization at the indicated positions (glutamine substitutions) and plus indicate presence of charges (Arg or Lys) on IS4, IIIS4 and IVS4. **b** Left panel shows representative families of *I*_Ba_ through wild type channel and through a channel construct where all IIS4 charges were neutralized (IIS4_N_). Barium currents were evoked during depolarization starting from − 40 with 10 mV increments from a holding potential of − 100 mV. Right panel shows representative tail currents. Currents were activated during a 20-ms conditioning depolarization to 0 mV. Deactivation was recorded during subsequent repolarizations with 10 mV increments starting from − 100 mV. **c** Left panel, averaged activation curves of wild type and IIS4N channels. Right panel, voltage-dependent time constants of channel activation/deactivation. Adapted from *Pflügers Archiv – European Journal of Physiology*. Beyl et al. [13]. © The Authors

Studies with CaV1.2 constructs where S4 charges were partially or completely neutralized provided further insights: If IIS4 and IIIS4 each contributed about 40% of the energy required for channel opening [71], then neutralization of all IIS4 charges is expected to affect CaV1.2 activation. Beyl et al. [13] observed the opposite of what was expected: Neutralization of all IIS4 charges resulted in a channel construct (IIS4N) which opened and closed with kinetics similar to wild type (Fig. 7). This suggested at first glance that IIS4 is hardly participating in gating (but see Fig. 10).

**Fig. 8:**
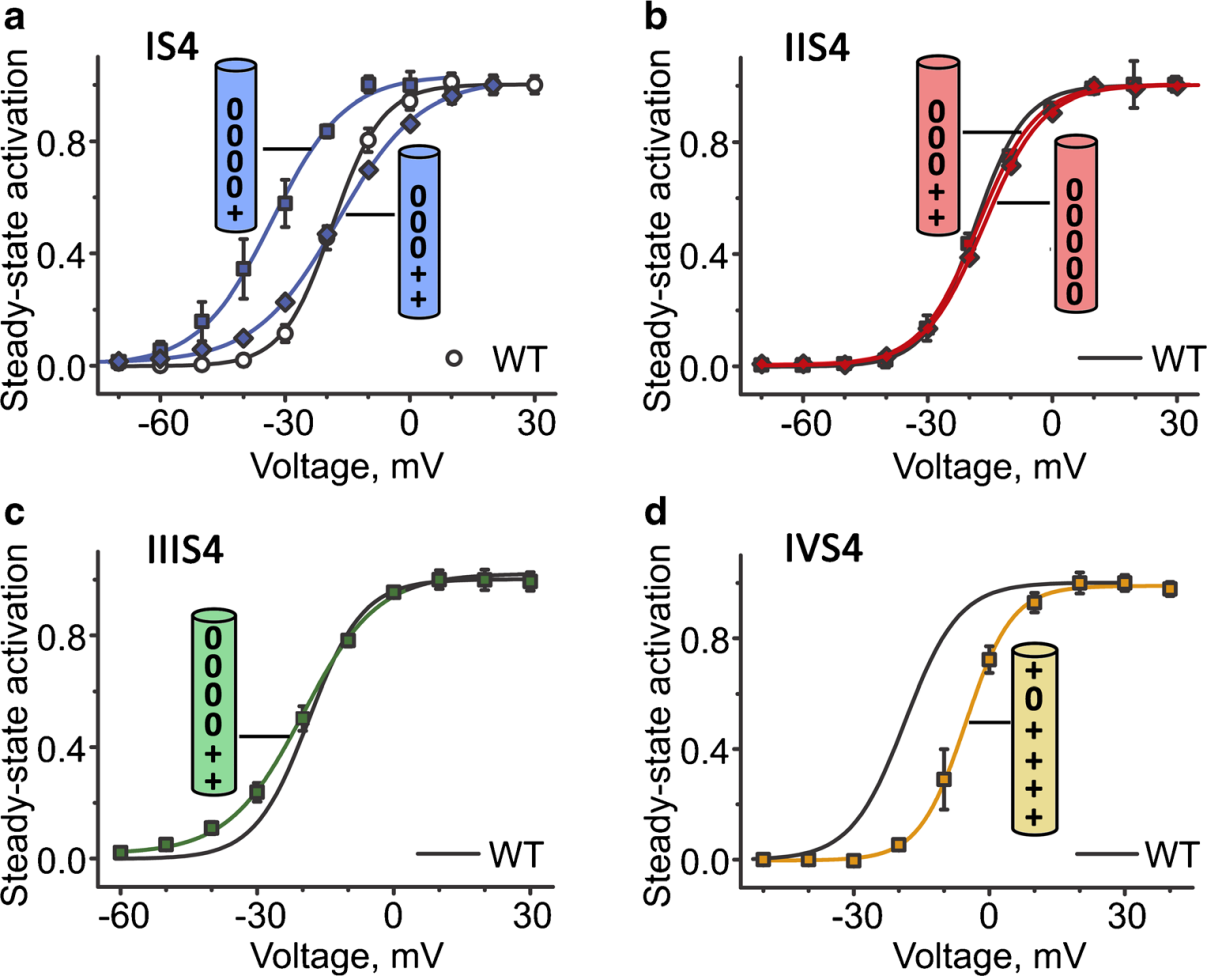
Key role of IS4 in activation gating: Movement of IS4 represents a rate-limiting stage. The cylinders represent the S4 residues with the positions of the mutations (plus stands for Arg or Lys, zero stands for Gln). **a**–**d** Averaged activation curves of wild-type and charge neutralization in IS4 (**a**), IIS4 (**b**), IIIS4 (**c**) and IVS4 (**d**). The most prominent changes in the slope of were observed for IS4 (**a**) and IIIS4 (**c**). Figure modified from *Pflügers Archiv – European Journal of Physiology*. Beyl et al. [15]. License: http://creativecommons.org/licenses/by/4.0/

In contrast, neutralization of IS4 (and to a lesser extent IIIS4) significantly decreased the slope of the steady state activation curve (Fig. 8). Calculating the effective charge from the slope of the Boltzmann distribution revealed that IS4 and IIIS4 carry most of the effective charge required for channel activation [15]. We conclude that the upward movement of segment IS4 (and IIIS4) is rate-limiting for releasing the pore gates (Fig. 8). A crucial role of IS4 and IIIS4 in CaV1.2 activation is in line with early work of Garcia et al. [41] and Yamaguchi et al. [109]. Remarkably, activation of IS4 in CaV3 was proposed to represent a rate-limiting stage in activation suggesting a general role of this segment in CaV channel gating [55]. Neutralization of single IVS4 charges has no significant effect on the slope (Fig. 8). The role of IVS4 remains currently unknown, because most constructs with more than one charge neutralized were not functional.

The lack of kinetic effects of IIS4 and IVS4 neutralization does not exclude their participation in gating but is likely to be obscured by the rate-limiting IS4 movement.

## Coupling points between VS and channel pore

**Fig. 9:**
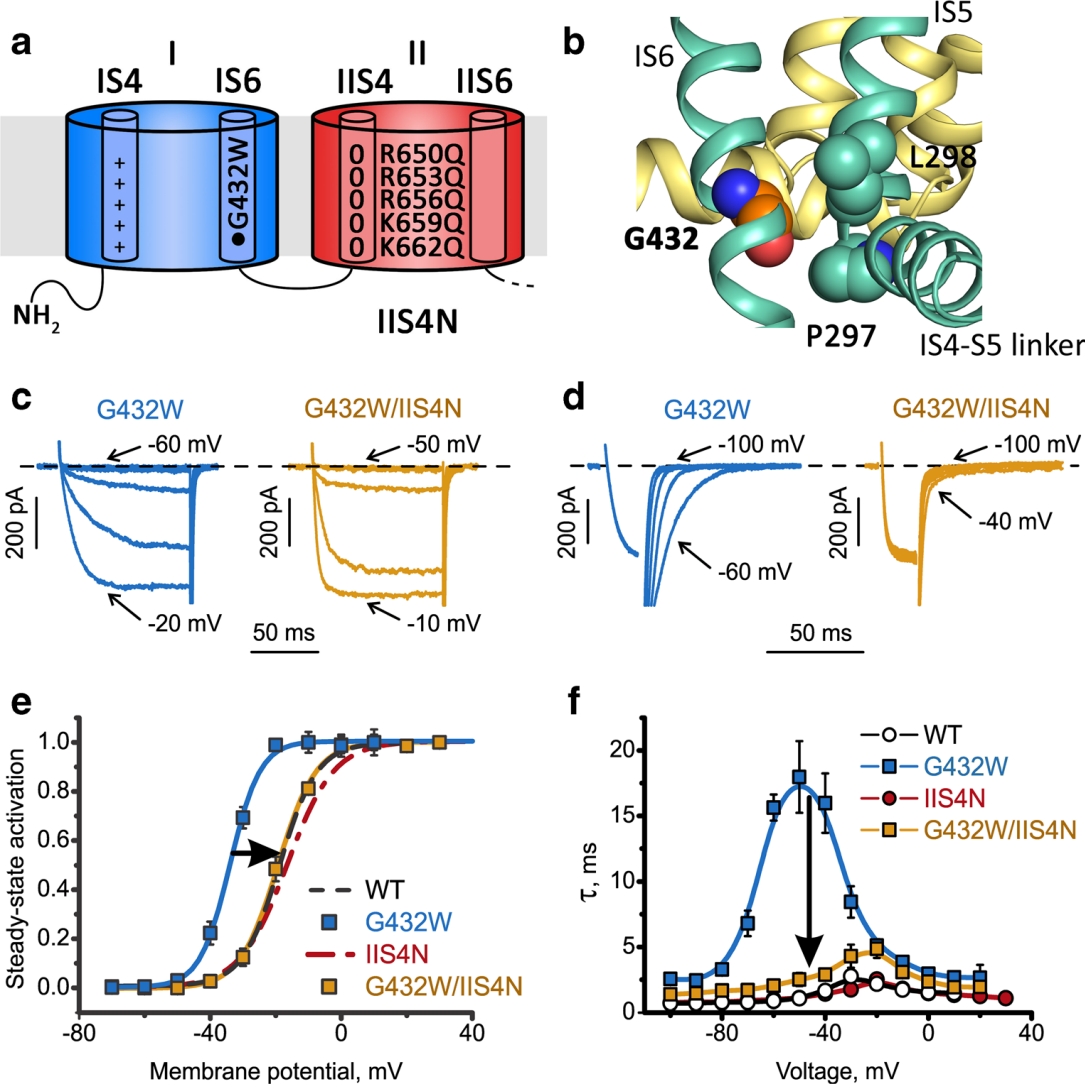
Slowly gating IS6 mutant G432W reveals role of segment IIS4: Neutralization of IIS4 accelerates current kinetics and shifts activation curve. **a** Schematic representation of α1 subunit domains I and II. The cylinders inside represent the S4 or S6 helices. Arg or Lys are shown as (+) while zeros (0) indicate charge neutralizations by Gln. Mutation G423W is highlighted on IS6. **b** Detailed view of position G432 of CaV1.2. The protein is represented as cartoon. Domain I is shown in green and domain IV in yellow. Glycine 432 is represented as orange spheres and the side chains of the closest residues (P297 and L298, both within 3 Å) are labeled and illustrated as green spheres. These two residues are located at the loop between the S4–S5 linker and the S5 helix. It shows tight packing of this gating sensitive area. This might indicate that even minor changes of the packing can lead to changes in the gating behavior due to necessary rearrangements within this region. For an overview on this position in other channels, see Supplementary Fig. 2. **c**–**f** Neutralization of all IIS4 charges (IIS4N) shifts the activation curve of a slowly gating IS6 mutant G432W in the depolarizing direction and accelerates current kinetics (arrows in **c** and **d**). Rightward shifts of the activation curves and acceleration of current kinetics by S4 neutralizations are exclusive for mutations in G/A/G/A positions (Beyl et al. [13]). **c**, **d** Representative currents through G432W and G432W/IIS4N highlighting slow activation (**c**) and deactivation of G432W and accelerated activation and tail currents in G432W/IIS4N (**d**). **e**, **f** Averaged activation curves (**e**) and voltage dependence of the activation/deactivation time constants (**f**) of WT, G432W, IIS4_N_, and G432W/IIS4N. **c**–**f** Adapted from *Pflügers Archiv – European Journal of Physiology*. Beyl et al. [13]. © The Authors

VSs and the pore are coupled. S4–S5 linkers directly interact with all four S6 segments via **G**432 (IS6), **A**780 (IIS6), **G**1193 (IIIS6), and **A**1503 (IVS6) designated as the **G/A/G/A** ring (exemplified for domain I in Fig. 9b, [15, 36, 103], see also Fig. 12a). Quantification of current kinetics revealed that mutations of G/A/G/A residues affect the movement of the VSs. A VS equilibrium constant Kvs = X(0)/Y(0) (see Fig. 3) increased in these mutants between 6- and 45-fold compared to wild type [13]. Effects on the VSs were exclusively caused by mutations of G/A/G/A residues and not observed for mutations of residues in neighboring positions (Table 1 in Beyl et al. [13]). Remarkably, G403R in CaV1.3 (corresponding to position G432 in CaV1.2) [84], A749G in CaV1.3 (corresponding to position A780 in CaV1.2) [77], as well as G369D in CaV1.4 (corresponding to position G432 in CaV1.2) [48] all cause channelopathies, highlighting the key role of these residues in channel gating of different CaV families (see Table 1).

## G/A/G/A mutants unravel VS movements

While complete charge neutralization of segment IIS4 CaV1.2 did apparently not affect current kinetics (Fig. 7), the situation changed dramatically when S4 charges were neutralized in combination with any of the G/A/G/A mutations. This is exemplified in mutation G432W(IS6) combined with a partially or fully neutralized IIS4 segment (Fig. 9). Mutation G432W shifts the activation curve to the left (Fig. 9e) and strongly decelerates the activation/deactivation (Fig. 9f). Neutralization of all IIS4 charges (examplified for G432W/IIS4N) shifted the curve back to the right and accelerated current kinetics (Fig. 9f).

Similar observations were made for various combinations of A780T(IIS6), G1193T(IIIS6), or A1503G(IVS6) with either IS4, IIS4, or IIIS4 neutralizations. Remarkably, any given G/A/G/A mutation was affected by any of S4 neutralization in either domains I, II, or III [13] (illustrated in Fig. 12a).

At least four conclusions can be drawn: First, each individual S4 segment modulates a concerted ensemble of four tightly interacting (interlinked) S6 segments and is thus not restricted to the S6 gate of its domain (Fig. 12b, “cooperative gating model” [13]). Second, neutralization of charges in segments IS4–IIIS4 reduces the stability of the open state (evident from accelerated deactivation, exemplified in Fig. 9e). Third, even a single charge on S4 enables its movement to a down position. Fourth, not all four VSs are obligatory for CaV1.2 deactivation. Hence, IIS4N evidently does not reach its down position but this does not prevent channel deactivation (Figs. 7 and 9, see also [51]).

## Stabilization of the open state requires all S4 charges

**Fig. 10:**
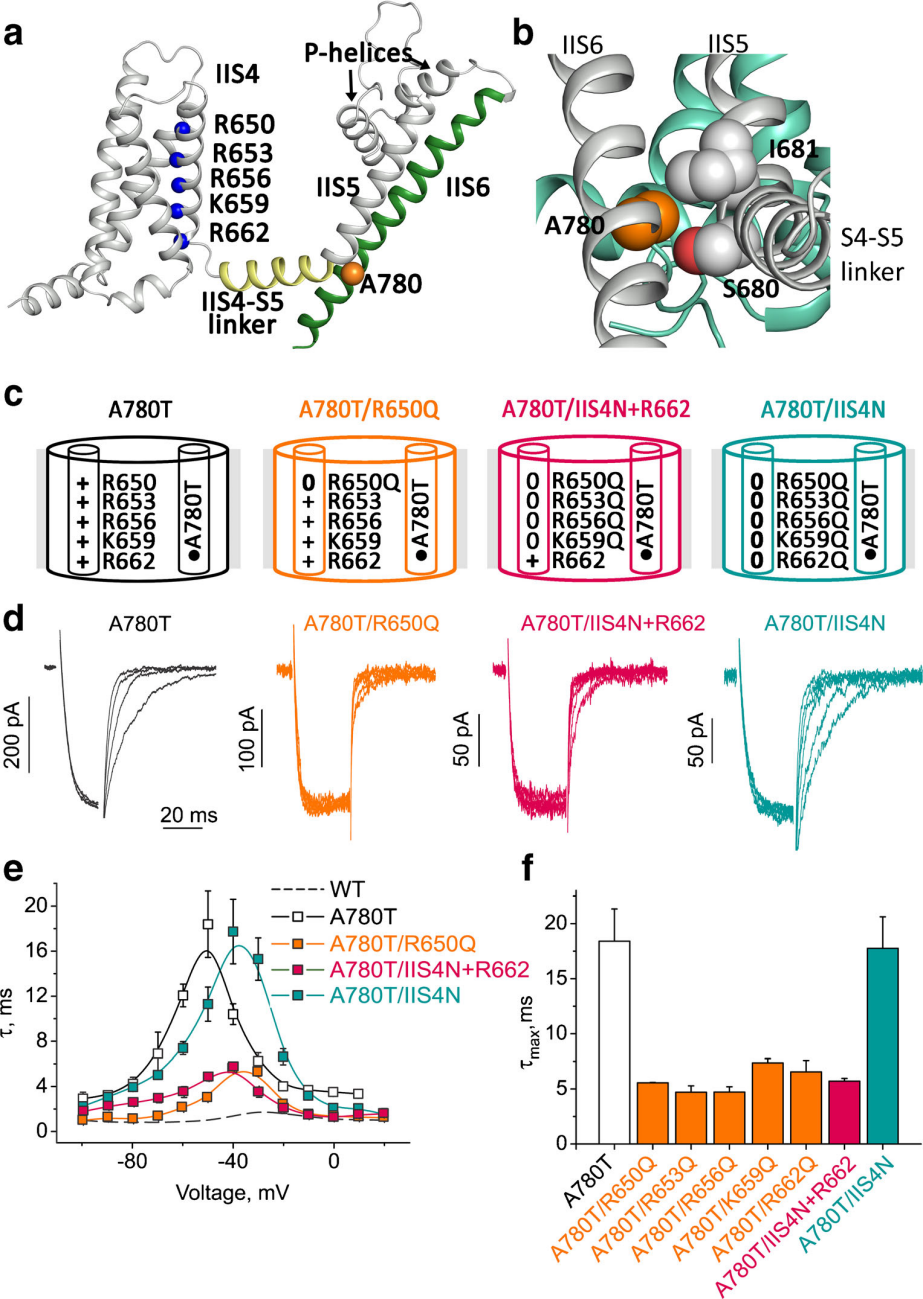
Full or partial neutralization of IIS4 prevents stabilization of the open state. Various IIS4 charge neutralizations (with exception of IIS4N) have similar gating effects on the slowly gating construct A780T. **a** A780 (orange sphere), like other G/A/G/A residues (Figs. 9b and S2 supplemental materials), putatively interacts with the S4–S5 linker and S5 loop. Positions of the charged residues on S4 are represented as blue spheres. **b** Detailed view of the position A780 of CaV1.2. Domain I (green) and domain II (gray). The side chain of A780 and the closest residues (S680 and I681, both within 3 Å) are represented as orange and gray spheres, respectively. S680 and I681 are located at the loop between the S4–S5 linker and the S5 helix. Similar to Fig. 9b, this shows the tight packing of the residues at this critical location. Changes in size or property of the A780 are likely to lead to changes in the dynamic behavior of the channel since this perfect arrangement can be important for the stabilization of certain states. **c** Schematic representation of the mutations in domain II. The cylinders inside represent the IIS4 and IIS6 helices. Plus stands for Arg or Lys, zero stands for Gln. **d** Currents of the indicated channel constructs (colors correspond to the illustrations in **c**). Channels were activated by conditioning pulses from − 80 to 10 mV, deactivation was induced by hyperpolarizing steps to voltages between − 70 and − 100 mV. **e** Voltage dependence of time constants of activation/deactivation. **f** Bar graphs illustrating the maxima of the bell shaped curves (shown in **e**) reflecting the slowest kinetics of activation/deactivation

Neutralization of even a single charge prevents the corresponding S4 segment from contributing to the stability of the open state irrespective of its position on the S4 helix. This is exemplified for mutant A780T, in which all five IIS4 charges were replaced by glutamine one by one (Fig. 10). Removal of any one of these single IIS4 charges shifts the activation curve rightwards and accelerates the current kinetics reflecting a reduction of open state stability [15]. We speculate that partially neutralized S4 segments are retained in a hypothetical “intermediate” state where they are unable to contribute to open pore stability.

## A single charge is sufficient for S4 contribution to pore closure

Complete neutralization of S4 charges (IIS4N, Fig. 10) makes this segment voltage-independent and thus prevents any pushing force on S6 towards its closing position. However, when a single charged amino acid remains on IIS4, this segment facilitates channel closure (evident from accelerated deactivation kinetics in A780T/IIS4N+K662, Fig. 10f).

Conformational changes in an S4 segment carrying only one elementary charge may be understood when we consider that a membrane voltage of 100 mV across a membrane with a thickness of 100 Å corresponds to tremendous 100 kV/cm (which is about an order of magnitude larger than the 14 kV/cm which causes a lightning discharge in air during a thunder storm).

## Voltage sensors have individual impacts on voltage-dependent inactivation

The upward movement of S4 segments during a membrane depolarization enables not only conformational rearrangements at the inner S6 helix bundle (resulting in pore opening, Fig. 3) but also causes a conformational change called voltage-dependent “inactivation.” Voltage-dependent inactivation of CaV channels is evident from the current decay with increasing membrane depolarizations. Inactivation in CaV channels is usually investigated with barium as the charge carrier to avoid the development of calcium-dependent inactivation (see [49] for review).

**Fig. 11:**
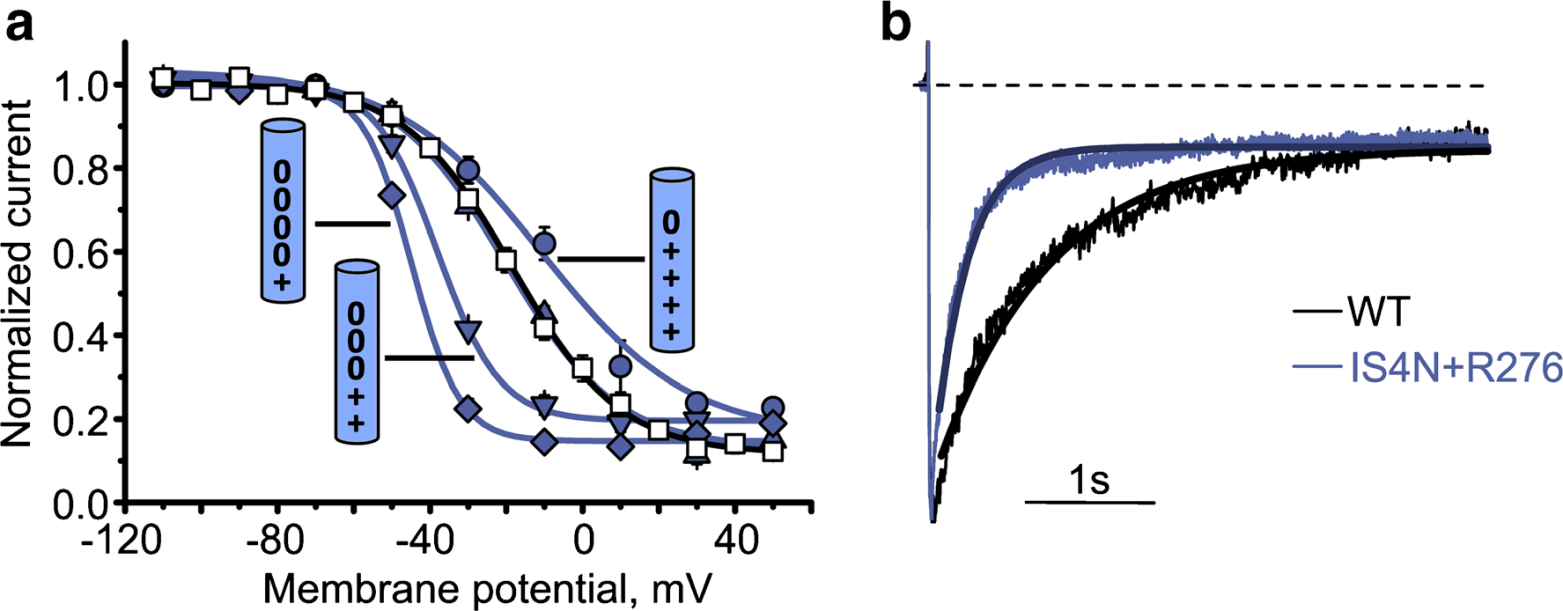
Key role of IS4 in inactivation gating: Neutralization of segment IS4 modulates CaV1.2 inactivation. **a** Steady state inactivation curves of WT and the indicated IS4 mutants. Slope of the Boltzmann curves ranged from 6.2 ± 0.7 mV in IS4N+R276 (*diamond*) to 17.4 ± 3.5 mV in K264Q (*circle*). The cylinders represent the S4 residues with the according mutations (plus stands for Arg or Lys, zero stands for Gln). **b** Superimposed typical normalized IBa through WT and mutant IS4N+R276. During 3s depolarizations from − 80 mV to the voltages of the maximum of the current–voltage relationship. Note the faster development of inactivation in IS4N+R276. Current decay was fitted to a monoexponential function yielding time constants of *τ*_inact_(WT) = 393 ± 24 ms and *τ*_inact_(IS4N+R276) = 235 ± 29 ms, respectively. Solid lines represent the fitted functions. Figure modified from *Pflügers Archiv – European Journal of Physiology*. Andranovits et al. [6]. License (http://creativecommons.org/licenses/by/4.0/)

Although voltage-dependent inactivation is likely to involve structural changes at the outer channel mouth near the selectivity filter [29–31, 56, 80], a number of point mutations in pore lining S6 and adjacent segments of CaV α subunits have been shown to modulate this process [46, 49, 93]. This includes a cluster of hydrophobic residues located close to the inner channel mouth on IS6 and IIS6 [36, 50, 58]. Other key inactivation determinants in CaV channels have been identified in intracellular loops [1, 46, 88, 92, 76]. Changes in inactivation caused by S6 mutations on CaV channels may be very substantial: a 75-fold acceleration of inactivation by a single point mutation was reported for M1811Q on IVS6 of Cav2.1 [12], while the Timothy syndrome mutation G402S in CaV1.2 prevents voltage-dependent inactivation almost completely [89]. Andranovits et al. [6] identified a key role of segment IS4 in voltage-dependent inactivation. Impairing IS4 function by charge neutralization had the largest and regular (charge-dependent) effects on voltage-dependent inactivation (Fig. 11), compared to no or less impact of equivalent charge neutralizations in segments IIS4 and IIIS4.

**Fig. 12:**
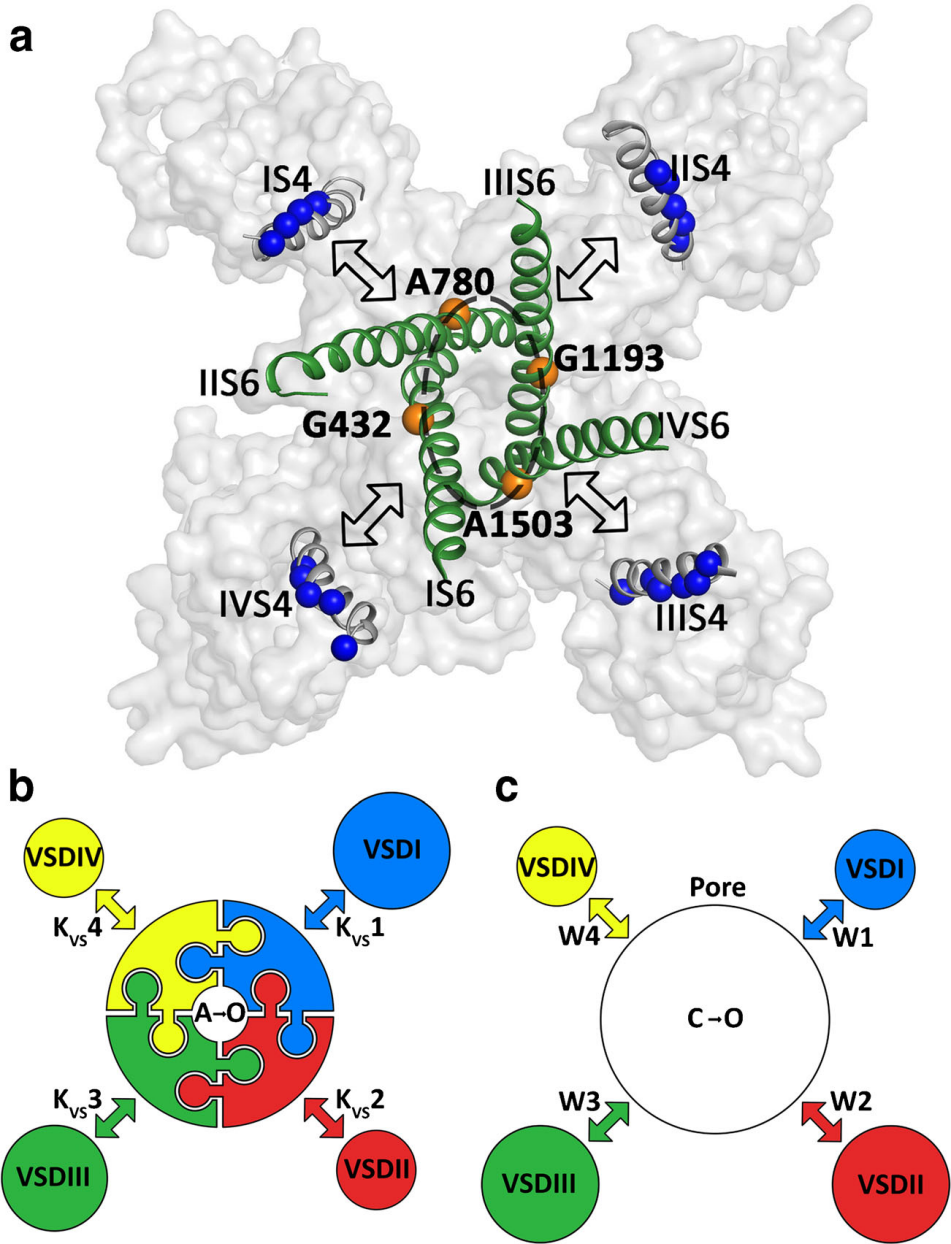
Structural determinants (**a**) and models (**b**, **c**) of CaV1.2 activation. **a** The α1c subunit in top view, represented as gray transparent surface. The S4 and S6 helices are shown as cartoon. Blue spheres represent the charged residues on the S4 segments. Orange spheres represent the G/A/G/A positions on the S6 segments. The black dotted line indicates a cooperative unit. The outlined double arrows indicate interactions of S4 segments (IS4 - IVS4) with all four G/A/G/A positions. Two gating concepts have been proposed: In the cooperative gating model (Beyl et al., figure **b**) upward movement of S4 segments disengages the interlinked (illustrated as invaginations) S6 gates and channels enter the activated/not open conformation. Further separation of S6 gates results in concerted channel opening. In this way, a single VS can modulate all four pore-forming elements (as observed in experiments [13]). Upward movement of IS4 (and IIIS4) is a rate-limiting step for activation [15]. **c** In a gating model proposed by Pantazis et al. [71], the authors estimated the energy (W1 to W4) contributed by individual S4 segments to pore opening based on fluorescence changes of CaV1.2 constructs with individually labeled S4 segments during voltage clamp steps. Calculated different energies (W1 to W4) suggest that IIS4 and IIIS4 provide most of the energy for pore opening. This figure is modified from *Proceedings of the National Academy of Sciences*. Pantazis et al. [71]

A gradual reduction of the slope factor of the inactivation curve on stepwise neutralization of IS4 charges is shown in Fig. 11. Apparent enhancement of inactivation caused by IS4 neutralization is also evident from the accelerated time course of current decay. This steeper voltage dependence (Fig. 11a) can be understood assuming that IS4 moves through sub-states that are stabilized by interactions of IS4 charges with surrounding residues [32]. In this scenario, neutralization of these charges reduces the number of interactions (e.g., salt bridges) and correspondingly the number of sub-states that IS4 can occupy between full activation and inactivation ([19], see also supplemental Fig. 2 in Andranovits et al. [6]).

## Conclusions and outlook

- Upward movement of S4 segments (at depolarization) is almost voltage-independent while downward movement (at hyperpolarization) is strongly voltage dependent. This suggests that upward movement is driven by intramolecular forces while downward movement is driven by membrane potential acting on the VS. Even a single charge is sufficient for downward movement of the S4 segment.
- S4 segments in their down position lock the pore gates in the closed state. Gates are unlocked only when all four segments leave the down position. Movement of IS4 (and IIIS4) is rate-limiting.
- S4 segments move to the up position via sub-states where their positively charged residues interact with residues of the “charge transfer centre.” Neutralization of any S4 charge (i.e., replacement by neutral glutamine) prevents its arrival in a final up position (“like in a broken zipper”).
- A ring of small residues (G/A/G/A ring) on the lower third of S6 segments interacts with the S4–S5 linkers. Constructs carrying mutations in these positions are extraordinary sensitive to charge neutralization, making them interesting tools for studying electro-mechanical coupling.
- Only a completely (fully) charged IIS4 contributes to the stability of the open state. In other words, the specific number of S4 charges (between 5 in IS4 and IIS4 and 6 in IIIS4 and IVS4) is likely to be essential for a given stability of the open state. We speculate that evolution effectively titrated the number of S4 charges (and interacting negative counter charges) to fine tune calcium entry.
- Future studies on quantitation of inactivation will have to answer the principle question: From which state (R, A, and/or O) does the majority of channels enter the inactivated conformation?

## Electronic supplementary material

ESM 1 (PNG 278 kb)

High resolution image (TIF 25514 kb)

ESM 2 (PNG 325 kb)

High resolution image (TIF 24521 kb)

ESM 3 (PNG 173 kb)

High resolution image (TIF 11042 kb)

ESM 4 (PNG 8771 kb)

High resolution image (TIF 11749 kb)

ESM 5 (DOCX 20 kb)
