# Supplementary figures and images for: Calcium channel gating
Source: Pflugers Arch. 2018 Jun 27;470(9):1291–309. doi: 10.1007/s00424-018-2163-7 (PMC6096772; doi:10.1007/s00424-018-2163-7)

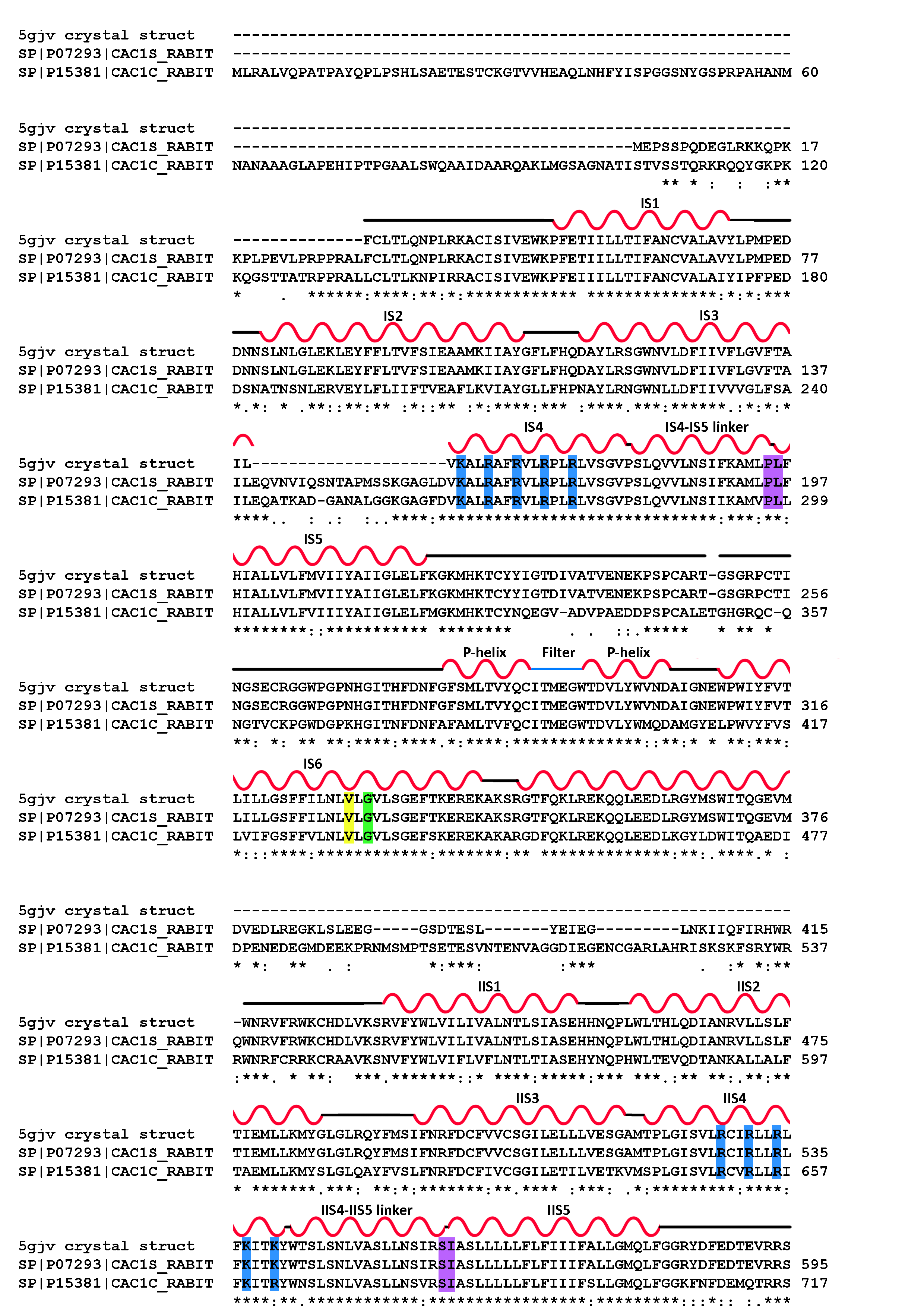

Supplement: Supplementary file 1 — (PNG 278 kb) [file 424_2018_2163_FIg13_ESM.png]

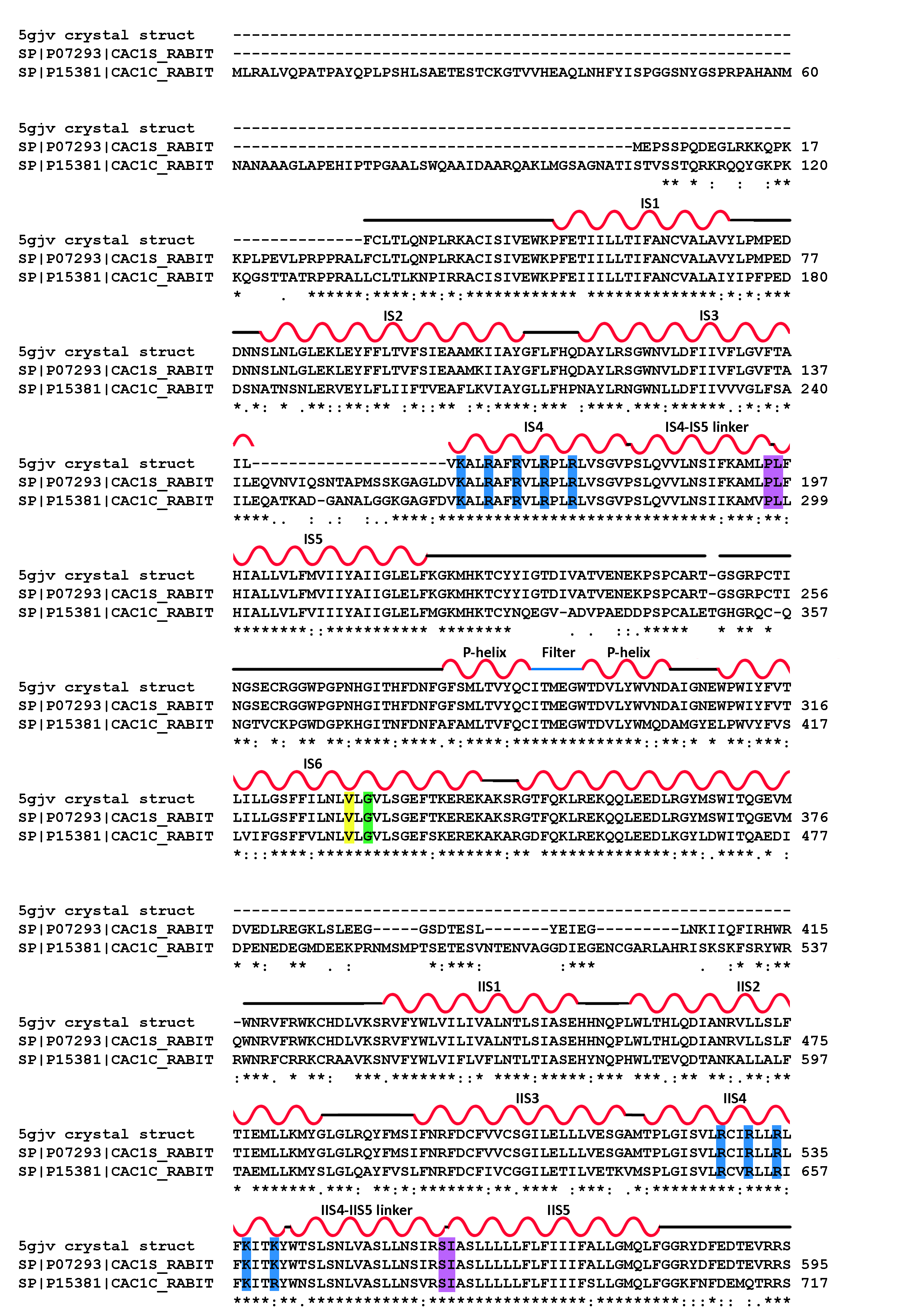

Supplement: Supplementary file 2 — High resolution image (TIF 25514 kb) [file 424_2018_2163_MOESM1_ESM.tif]

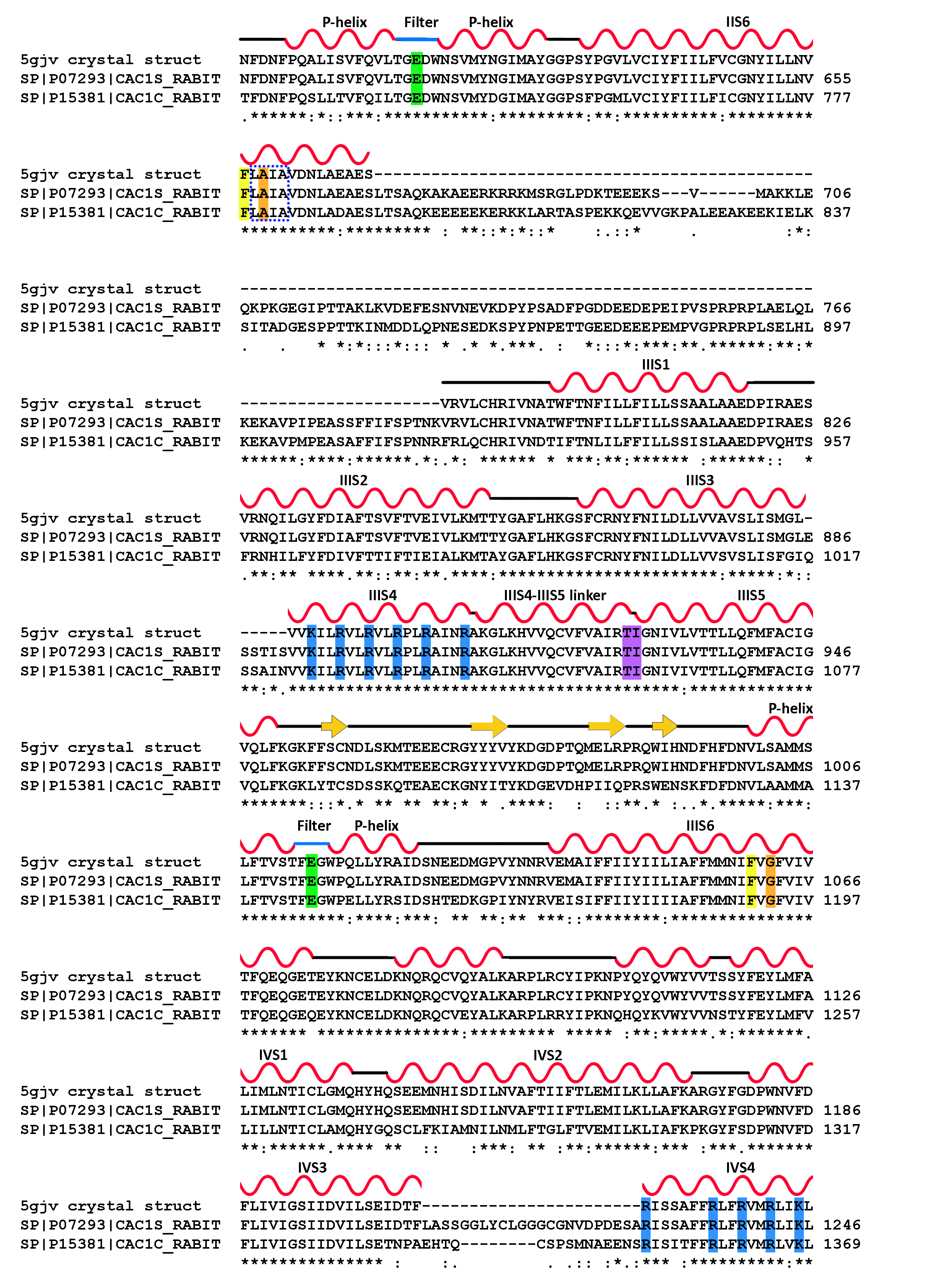

Supplement: Supplementary file 3 — (PNG 325 kb) [file 424_2018_2163_Fig14_ESM.png]

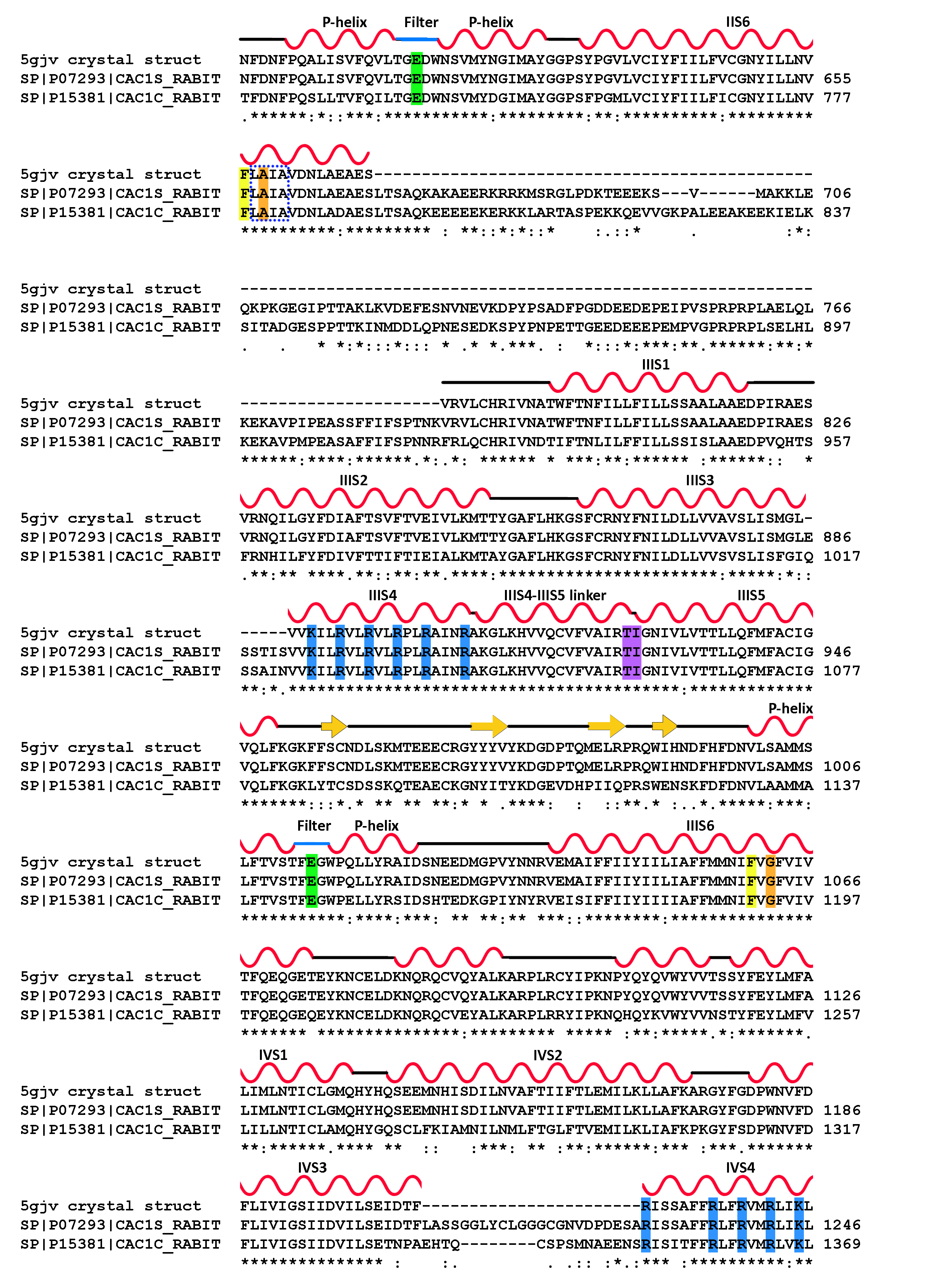

Supplement: Supplementary file 4 — High resolution image (TIF 24521 kb) [file 424_2018_2163_MOESM2_ESM.tif]

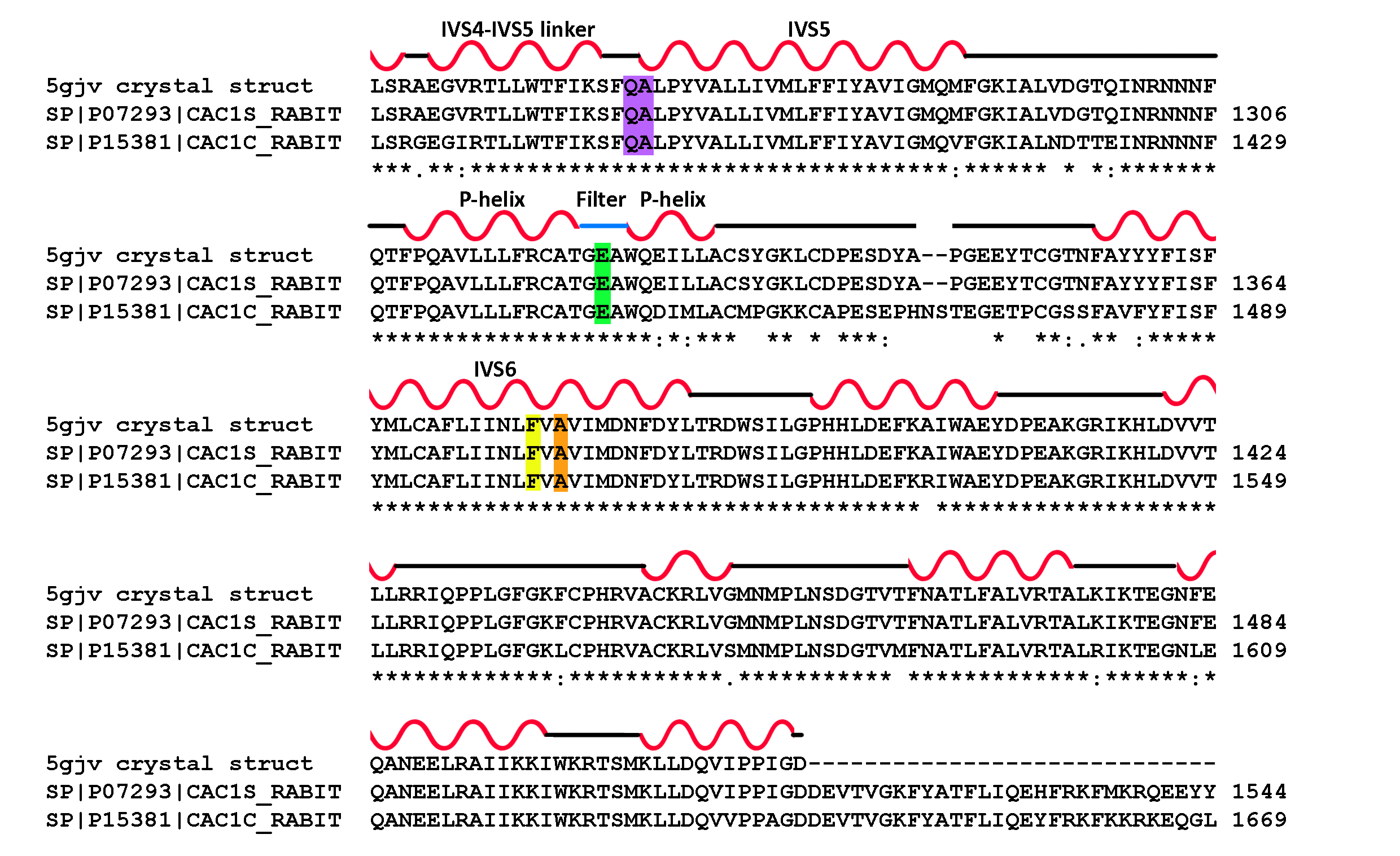

Supplement: Supplementary file 5 — (PNG 173 kb) [file 424_2018_2163_Fig15_ESM.png]

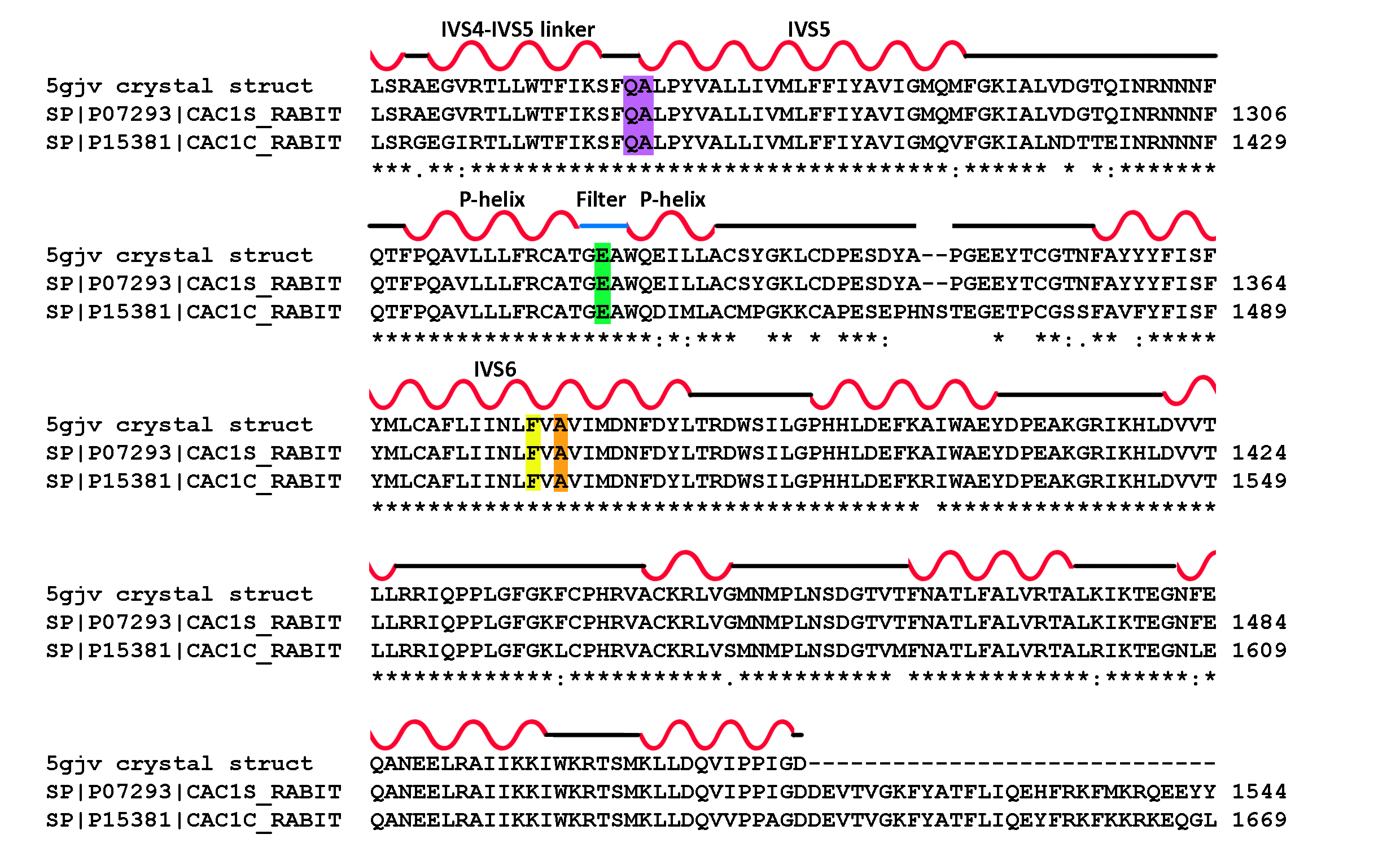

Supplement: Supplementary file 6 — High resolution image (TIF 11042 kb) [file 424_2018_2163_MOESM3_ESM.tif]

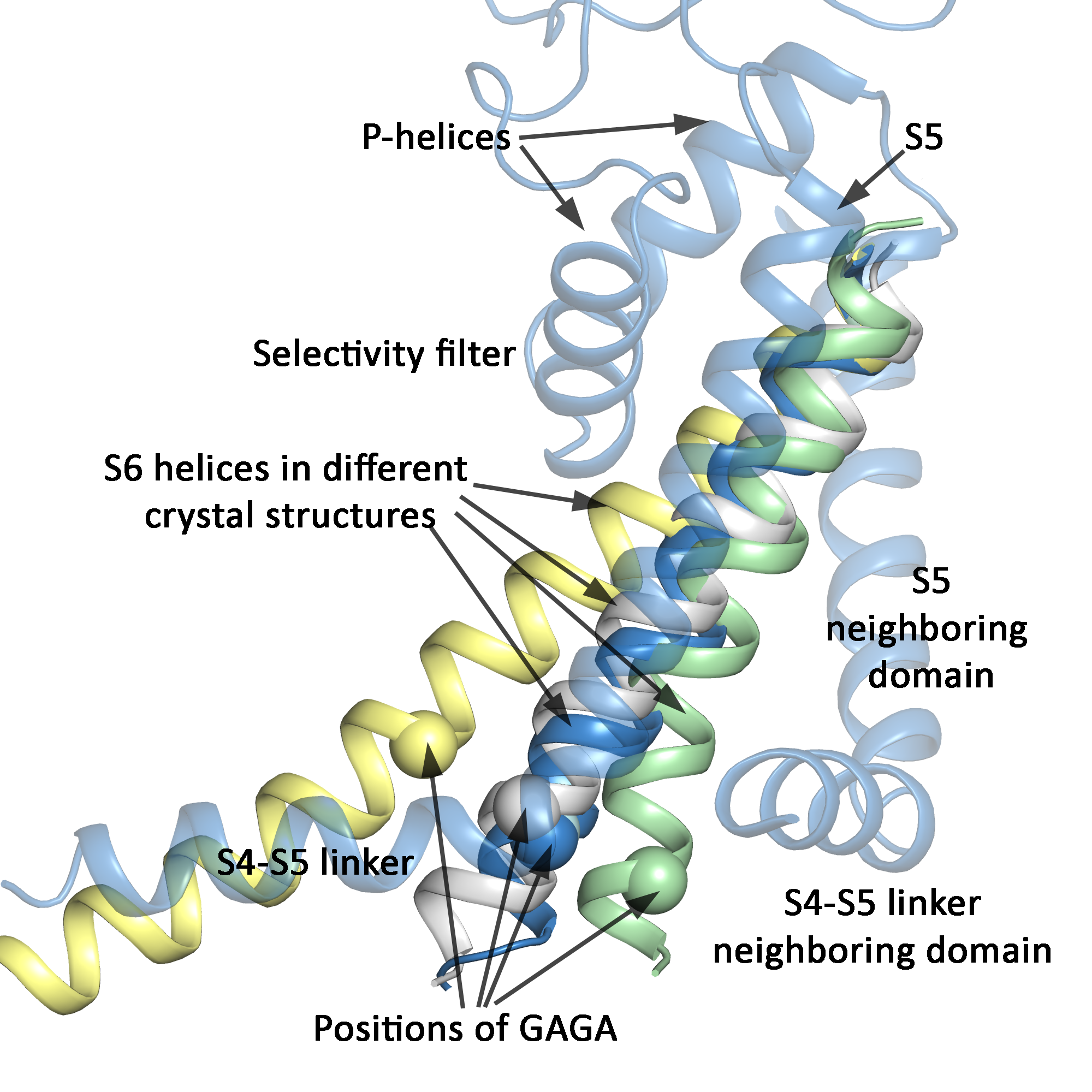

Supplement: Supplementary file 8 — High resolution image (TIF 11749 kb) [file 424_2018_2163_MOESM4_ESM.tif]
