# Supplementary material for: Calcium channel gating
Source: Pflugers Arch. 2018 Jun 27;470(9):1291–309. doi: 10.1007/s00424-018-2163-7 (PMC6096772; doi:10.1007/s00424-018-2163-7)
Supplement: Supplementary file 9 — (DOCX 20 kb) [file 424_2018_2163_MOESM5_ESM.docx]

**Supplemental material**

**Calcium channel gating**

**Pflügers Archiv - European Journal of Physiology**

**Hering S.^†^, Zangerl-Plessl E.-M., Beyl S., Hohaus A., Andranovits S., Timin E.N.**

**^†^**Corresponding author: Steffen Hering, e-mail: steffen.hering@univie.ac.at, phone: +43-1-4277-55310; Department of Pharmacology and Toxicology, University of Vienna, Althanstrasse 14, 1090 Vienna, Austria

***Supplementary Fig. 1 Sequence alignment of the α–subunit of CaV1.1 (cryo–EM construct*** [100] ***and channel sequence) and CaV1.2***

*The sequence identity line corresponds to the identity between CaV1.1 (P07293, rabbit) and CaV1.2 (P15381, rabbit), not the cryo–EM structure (5gjv_crystal_struct). Helices S1 to S6 as well as the selectivity filter are indicated according to the cryo–EM structure. Residues discussed in this review are highlighted as follows: The positively charged residues of the S4 helices are highlighted in blue, the EEE motive of the filter in green, the fenestration points from the cavity towards the intracellular side in yellow, the G/A/G/A residues in orange and the residues in close contact with the G/A/G/A residues in the S4–S5 linker loops in purple.*

***Supplementary Fig. 2 Hypothetical role of G/A/G/A positions in different calcium and sodium voltage gated ion channels***

*Structural alignment of the pore–helices as well as the selectivity filter of different calcium and sodium channels. For better visibility, only the CaV1.1 crystal structure (pdb–code: 5gjv) pore domain is represented in full in blue, the remaining structure except for the S6 helix is set to transparent. For the other crystal structures only the S6 are shown for clarity. The yellow S6 helix is from the open NaVMs structure by Sula et al* [91]*. The grey S6 represents the recently published EeNaV1.4 by Yan et al,* [104]*. The green S6 constitutes the closed conformation of the NaVAb structure by Lenaeus et al* [59]*. The spheres indicate the Cα of the G/A/G/A position. This indicates that when the S6 is closest to the S4–S5 linker and its loop to the S5, the G/A/G/A position faces towards this loop and is involved in a region of tight packing. This could indicate that throughout the transition from the closed to the open channel* these specific residues experience a closer contact with this region than their neighbouring positions and therefore might be crucial for the dynamic process of channel opening and closing.
